# “That was one of my most difficult and biggest challenges”: experiences, preconditions and preventive measures of health-oriented leadership in virtual teams – A qualitative study with virtual leaders

**DOI:** 10.1186/s12889-024-18800-7

**Published:** 2024-05-17

**Authors:** Ilona Efimov, Volker Harth, Stefanie Mache

**Affiliations:** Institute for Occupational and Maritime Medicine, University Medical Center Hamburg-Eppendorf, Seewartenstr. 10, 20459 Hamburg, Germany

**Keywords:** Leadership, Leader–employee interaction, Remote work, Teleworking, Working conditions

## Abstract

**Background:**

Health-oriented leadership (HoL) has a positive impact on health- and work-related outcomes of employees in face-to-face settings. Increased digitization during the COVID-19 pandemic has led to many changes and increased job demands. According to current state of research, HoL in virtual teamwork is insufficiently researched. The aim of the study is to examine the experiences of virtual leaders during the COVID-19 pandemic and to identify preconditions and preventive measures for promoting HoL.

**Method:**

Using a qualitative study design, semi-structured, guide-based telephone interviews were conducted with 16 German virtual leaders between May and July 2021. The collected data were inductively analyzed and interpreted using qualitative content analysis according to Mayring. Explorative analyses of differences between leaders with and without pre-pandemic experiences with virtual leadership were made.

**Results:**

Results indicated that leaders, regardless of pre-pandemic experiences with virtual leadership, faced diverse challenges in implementing HoL in virtual teamwork during the COVID-19 pandemic. Virtual leaders perceived personal preconditions (e.g., leaders’ characteristics or behaviors), organizational preconditions (support by management or open-minded corporate culture), social preconditions (e.g., social support by team) and technical preconditions (e.g., sufficient technical equipment) as conducive to implementation of HoL. Almost all leaders with pre-pandemic experience identified a need for structural preventive measures, whereas almost all leaders without pre-pandemic experience reported a need for behavioral preventive measures in order to promote HoL in virtual teams.

**Conclusions:**

This study suggests that implementing HoL in virtual teamwork is challenging, complex and requires adjustments in leadership behavior. Thereby, the study provides initial empirical findings for a holistic approach to HoL implementation in virtual teams, considering beneficial multilevel preconditions. Due to a limited generalization of present results, longitudinal and interventional studies will be necessary for the analysis of causal relationships in future research. In particular, a holistic research perspective in order to understand the complex, contextual interdependencies of leadership is recommended. In practice, based on a differentiated needs analysis, structural preventive measures for a holistic organizational development as well as behavioral preventive measures for ongoing personnel development are recommended.

**Supplementary Information:**

The online version contains supplementary material available at 10.1186/s12889-024-18800-7.

## Background

The COVID-19 pandemic led to a significant increase worldwide in the use of mobile work such as home offices. In Germany, the percentage of all employees working from home almost doubled compared to the pre-pandemic period: in 2019, the percentage was 12.8% of all employees, increasing to 24.8% in 2021 [1]. The numbers of employees working from home in 2021 varied greatly between sectors, with most employees in IT services (75.9%) and the fewest in health care (5.4%) [1]. In this regard, a study conducted in Germany in 2020 showed that of all surveyed employees who reported working from home permanently or on some days, about 45% had started doing so as recently as the COVID-19 pandemic [2]. The pandemic-related changes in collaboration led to increased job demands and impaired wellbeing for many employees [3], especially in the absence of organizational support [4]. A review further revealed that personal factors (e.g., personal living conditions or personal resources) or organizational factors (e.g., leadership style or job autonomy) influenced perceptions of remote or home office work rather than this working arrangement having intrinsically homogeneous consequences on employees [5]. Fewer studies looked at the impact of the pandemic on leaders. The latter also reported increased job demands (e.g., work intensification) and challenges in virtual or hybrid leadership [6–9]. The Global Leadership Forecast 2021, a global survey among leaders, found that 60% felt exhausted after each workday, 23% did not experience themselves as effective at leading virtual teams, and less than 30% received training in this area and accordingly felt less prepared [10]. The definition of virtual leadership for this study is based on [11].

Yet it is relevant that leaders have a good state of health and are skilled at leading in a healthy and effective way, as they may serve as a protective factor for employees in the face of work strain [12–14]: by means of their leadership behavior (direct influence), design of working conditions, their function as a role model and their own state of health, which in turn may influence leadership behavior (indirect influence) [15]. In contrast to established leadership concepts focusing on leadership behavior (such as transformational leadership) in relation to follower health [16], theoretical concepts of healthy leadership have also been developed over the last two decades [17]. A dominant concept is the “health-oriented leadership” (HoL) approach according to Franke and colleagues [16] which also serves as a theoretical framework for this study. This broader approach integrates leader and follower perspectives and differentiates between follower-directed health-oriented leadership (i.e., StaffCare) and self-directed health-oriented leadership (i.e., SelfCare). Thus, the theoretical model consists of three components: SelfCare of the leader, StaffCare and SelfCare of followers. Each of these components comprises three dimensions: value, awareness and behavior. The SelfCare of the leader forms the basis in this model. Thereby, leaders should consider their own health to be relevant, be able to perceive changes when exposed to stress factors, and lastly be able to apply appropriate health-promoting behaviors towards themselves. Subsequently, the SelfCare of the leader enables StaffCare and can positively influence (in a direct and indirect way) the SelfCare of the employees. In the corresponding instrument, the components can be assessed by both leaders and employees [16]. According to current research, this theoretical model has been predominantly studied in traditional, face-to-face work contexts, indicating a positive impact on follower health [18]. Initial quantitative studies on HoL in digital, remote collaboration during the COVID-19 pandemic indicated that high levels of SelfCare among leaders had a positive impact on, among others, their own health, well-being, and job satisfaction [9], and that high levels of StaffCare had a positive impact on employee (mental) health and wellbeing [19], also when compared to office-based collaboration [9, 20, 21]. Study results by Klebe and colleagues showed that ICT hassles weakened the positive relationship between StaffCare and employees’ mental health [19]. Furthermore, a qualitative interview study with virtual leaders before the COVID-19 pandemic found that applying HoL in virtual collaboration was possible, but required adjustments in StaffCare awareness and behavior, e.g., support in boundary management [22]. Another qualitative study on HoL and transformational leadership during the COVID-19 pandemic revealed that lack of social presence, limited informal chats, communication difficulties and lack of mutual trust was experienced by leaders and employees as challenging in the remote setting compared to the traditional office setting [23]. Overall, reviews demonstrated that there is insufficient evidence on leadership influence on followers’ mental health outcomes in digital, remote collaboration [11] and, in particular, in recent years few empirical studies on HoL in this context [18]. Researchers are currently arguing whether pre-pandemic research findings on virtual leadership and teamwork are transferable to the remote work context created by the COVID-19 pandemic [24–26]. In addition, previous studies did not analyze results on (healthy) leadership in digital, remote collaboration during the COVID-19 pandemic between individuals with and without prior experience – besides one study on perceived efficacy of virtual leadership between leaders with and without previous experience of working from home [27]. Therefore, it is currently an open question whether experiences of leaders who regularly worked in virtual teams before the COVID-19 pandemic differ from individuals who were forced to move their work to home offices ad hoc during the pandemic.

Furthermore, reviews have shown that leadership does not function in a vacuum, but rather exists in a context [28], requiring a systematic approach and an analysis of contextual conditions in the cause-effect relationship between virtual leadership and health outcomes [11, 29, 30]. However, many organizations were unprepared for the ad hoc changes in the workplace brought about by the COVID-19 pandemic [26]. Accordingly, different conditions and preconditions may have existed. Initial studies on conducive conditions of virtual leadership during the pandemic revealed the relevance of organizational and social support for leaders [8, 31]. Similarly, there are generally few studies on conducive conditions for HoL [11]. For the traditional, face-to-face work context, leaders’ own SelfCare [32], but also job resources related to social relationships (such as social support by colleagues [33], team health climate [34] or organizational health climate [35, 36]) as well as to work organization (such as high-performance work practices or health-oriented human resources management strategies [37]) were found to positively influence HoL. For the digital, remote work context, only one pre-pandemic qualitative explorative study assessed work characteristics influencing the application of HoL in virtual teams [22]. The findings indicated that, besides social, technical and personal factors, organizational factors (e.g., flexible working conditions, structural offers as well as a conducive management board and culture) were particularly relevant [22]. Accordingly, there is little evidence on preconditions of HoL in general, as well as in virtual teamwork or during the COVID-19 pandemic.

Summarizing all above listed research gaps, there is a lack of research on HoL in virtual teamwork in general as well as during the COVID-19 pandemic [18, 22] (also in relation to prior experience with virtual leadership) and on preconditions [36]. Thereby, this study aims to answer the following research questions:

- What are the experiences and challenges for (ad hoc) SelfCare and StaffCare in virtual teams in times of the COVID-19 pandemic?
- Which preconditions enable HoL in virtual teams?
- Which preventive measures may promote the use of HoL in virtual teams?
- How do leaders with and without pre-pandemic experience working in regular virtual or hybrid teams differ in their experiences and opinions?

## Materials and methods

### Study design

The present explorative interview study followed a qualitative research approach, which was assumed to be most appropriate for gaining initial insight in a new field of research [38]. Thereby, the study is based on the phenomenological approach as methodological orientation for accessing subjective perspectives [38, 39]. Between May and July 2021, a total of 16 semi-structured, guide-based telephone interviews were conducted with German virtual leaders. The use of telephone interviews was based on research economics, flexibility in time and place as well as geographical expansion of the sample [40]. The COREQ checklist (Consolidated criteria for reporting qualitative research) was used to ensure the quality of the methodology report in this study (see supplementary material 1) [41]. The first author (IE), a female psychologist (M.Sc.) and research assistant, conducted the study, from recruitment to data coding and interpretation, as part of her PhD thesis. She had practical experience in qualitative interviewing from previous research projects at the time of the study.

### Data collection

Applying the inclusion criteria, leaders were invited to voluntarily participate in this study who (1) worked mostly digitally at a computer workstation (e.g., 3 days/week), and therefore had practical experience with virtual leadership before and/or during the COVID-19 pandemic, (2) had at least 1.5 years of professional experience, (3) were German speaking and (4) at least 18 years old. Recruitment followed the snowball system through personal and professional contacts as well as professional networking platforms. In addition, recruitment took place via presentations at a scientific conference and an unpaid, free presentation for members of a business association. No personal relationship existed with any interviewee beforehand. All interviews were conducted in German. Study participants were informed about interviewer’s and study background, objectives, procedure, and data protection before study conduct. They were given the opportunity to take part in the interview flexibly in terms of time and place (from the company office or home office). Aiming at adopting an emic perspective [42], the problem-centered interview (PCI) method according to Witzel was used to conduct interviews [43]. The PCI method was considered to be particularly suitable as it aims at a joint understanding process between interviewer and interviewee and explores subjective experiences of interviewees (such as specific behavior, reasons, opinions) through dialogue. According to this method, four PCI instruments were employed: a short questionnaire for socio-demographic data, an audio tape recorder, an interview guide, and a postscript [43, 44]. At the beginning of the interview, the participants were also informed about the theoretical model [16] that was used as a framework for this study. In line with our research questions, interview participants were asked about their experiences and challenges of (ad hoc) implementing SelfCare and StaffCare in virtual teamwork during the COVID-19 pandemic, which preconditions affected them in doing so, and which preventive measures they considered supportive in promoting the use of HoL in virtual teams. The interview guide is displayed in supplementary material 2. A pre-test was conducted to evaluate both content related comprehension and time requirement. To the researcher’s knowledge, no other people were present during the interviews. Interviews length was 46 min on average (range between 35 and 66 min). No interviews were excluded or repeated. With the repetition of experience narratives by different participants, theoretical saturation was assumed over the course of the study.

### Data analysis and presentation

The collected, recorded data were transcribed verbatim (in terms of content and semantics [45]), anonymized, and inductively analyzed and interpreted using Qualitative Content Analysis by Mayring [46]. The characteristics of the sample were summarized in a descriptive manner (see Table 1). The coding of qualitative data was analyzed and classified into main and subcategories. The category structure was discussed in the research team until consensus was reached. The coding tree (see supplementary material 3) corresponds to the process model of summary [46]. In addition to summary analysis, two within-sample groups were created (leaders with and without pre-pandemic experience with regular virtual or hybrid teamwork) and results per categories were compared between these two groups in an exploratory manner. Study participants were not involved in data transcription or analysis. Data collection, transcription and analysis was conducted in German and results (e.g. interview quotes) were translated to English for the manuscript. For both analyses steps the software MAXQDA Plus for Qualitative Data Analysis (Version 20.0.6, 2020, VERBI GmbH, Berlin, Germany) was used.

### Ethical considerations

The study was conducted in accordance with the Declaration of Helsinki and approved by the Local Psychological Ethics Committee of the Hamburg Psychosocial Medical Center of the University Medical Center Hamburg-Eppendorf, Germany (LPEK-0240). All study participants received written information about the study beforehand and signed an informed consent form regarding the data collection, transcription and analysis of the interviews.

## Results

In the following, descriptive results on study participants’ characteristics as well as main results are presented. The main results are outlined based on four main categories: (1) Implementation of SelfCare in home office during COVID-19 pandemic, (2) Implementation of StaffCare in virtual teams during COVID-19 pandemic, (3) preconditions of HoL in virtual teams and (4) preventive measures for promoting HoL in virtual teams. See supplementary material 3 for our corresponding coding tree. Each main category is supplemented by the explorative comparison of two groups (leaders with and without pre-pandemic experience with regular virtual or hybrid teamwork). Participant quotations are presented to illustrate findings. For readability reasons, a selection of quotations is displayed in the manuscript text; further quotations can be found in supplementary material 4.

### Study participants’ characteristics

In this study sample (*N* = 16), most leaders were male (*n* = 11, 68.75%) and between 31 and 40 years old or between 51 and 60 years old (respectively: *n* = 6, 37.50%; mean age of 45.7 years). The majority was employed in the service industry (*n* = 11, 68.75%; of which *n* = 5, 31.25% were in IT / software) and in largescale companies (*n* = 12, 75.00%; of which *n* = 5, 31.25% were in companies with more than 10,000 employees). Almost all participants worked full-time and permanently (*n* = 15, 93.75%). About half of the study sample worked between 40 and 50 h per week (*n* = 9, 56.25), and 6 leaders reported working often or very often at irregular times, such as late evenings, nights, weekends, or vacations (37.50%). Most participants had leadership responsibility for a team (*n* = 8, 50.00%) or a department (*n* = 5, 31.25%), with a leadership span of less than 10 employees (*n* = 6, 37.50%) or greater than 26 employees (*n* = 7, 43.75%). The majority was operating at national level (*n* = 13, 81.25%), with team members most commonly located regionally (*n* = 10, 62.5%). Moreover, more than half of the participants (*n* = 9, 56.25%) indicated that prior to the pandemic, they either solely collaborated face-to-face with their team or, even though home office was occasionally used within some teams, the focus of collaboration was face-to-face. 7 leaders (43.75%), by contrast, were familiar with regular virtual or hybrid collaboration in their teams prior to COVID-19 pandemic (e.g., due to international geographical team distribution). Table 1 displays the characteristics of the sample.

**Table 1:** Study participants’ characteristics (*N* = 16)

Variable	*n*	%
**Gender**		
Male	11	68.75
Female	5	31.25
**Age**		
31–40 years	6	37.50
41–50 years	3	18.75
51–60 years	6	37.50
≥ 61 years	1	6.25
**Industry**		
Services	11	68.75
Logistics	3	18.75
Manufacturing	2	12.50
**Company size**		
≤ 49 employees	2	12.50
50–249 employees	2	12.50
≥ 250 employees	12	75.00
**Employment**		
Full-time (permanent)	15	93.75
Part-time (permanent)	1	6.25
**Working hours per week**		
30 h per week	1	6.25
40 h per week	3	18.75
40–50 h per week	9	56.25
> 50 h per week	3	18.75
**Work at irregular times**		
Never	1	6.25
Rarely	6	37.50
Sometimes	3	18.75
Often	5	31.25
Very often	1	6.25
**Job tenure**		
≤ 5 years	4	25.00
6–10 years	6	37.50
11–15 years	1	6.25
16–20 years	1	6.25
≥ 21 years	4	25.00
**Job position**		
Management	3	18.75
Head of department	5	31.25
Team lead	8	50.00
**Leadership span**		
≤ 10 employees	6	37.50
11–20 employees	3	18.75
≥ 26 employees	7	43.75
**Geographical team distribution**		
National (Germany)	13	81.25
International	3	18.75
**Experience with virtual or hybrid teamwork prior to COVID-19 pandemic**		
Yes	7	43.75
No	9	56.25

### Implementation of SelfCare in home office during COVID-19 pandemic

Overall, in terms of SelfCare in home office during the COVID-19 pandemic, a total of nine leaders reported on feasibility of implementation, but also on associated challenges.

#### Feasibility of implementing SelfCare

With regard to implementation of SelfCare in home office during the COVID-19 pandemic, a total of four leaders indicated that the implementation was feasible for them and that they had not experienced any challenges in this process. Some explained this by their flexible, autonomous and self-responsible working conditions.

*“Yes, it is absolutely possible for myself, since no one tells me how to organize my working time, I have full control over it. And everything that did not go well, I have to account for myself. And everything that worked out well, too. So, like everyone else, I’m learning how it can work.” [Participant #9, age 51–60 years, head of department]*.

#### Challenges for SelfCare implementation

However, four leaders indicated that SelfCare was feasible but challenging in home office. They reported that they have learned to manage stressful challenges (such as constant availability, limited opportunities to balance work due to longer working hours and fewer breaks) through self-reflection and self-discipline over the course of the pandemic. It was argued that virtual collaboration required more involvement, focus, and preparation.

*“Practicing self-care for oneself, of course, is a matter of self-discipline, and also partly a matter of organization at home, which is not always easy. I believe it is possible for oneself. […] So, in fact, I had to readjust and discipline myself when there were long periods at home. […] I also introduced rituals for myself, in order to consciously transition from professional to private life, so to speak.” [Participant #12, age 41–50 years, head of department]*.

In addition, one of those four leaders highlighted the need for constant self-reflection, since despite comprehensive knowledge on self-care, implementation remained difficult.

*“But then you also realize that you are honestly quite careless about your own person. So, this self-awareness and also perceiving myself when I’m moving in such a downward spiral, so to speak, and actually also exceeding my own limit, […] although one perceives it. That’s the issue of ‘interested self-endangerment’, something like that. We can all make a hook on it. We are not consistent enough, because we actually know that it is not okay at some points. And we regularly exceed these limits, and we communicate this to ourselves. It’s not even that we all don’t talk about it, but it’s often said: ‘Well, we have to somehow change that now,’ but still it doesn’t work (laughs). […] And, it has to be said, we are all managed appropriately. So, we are all, in the areas in which I am predominantly engaged, all senior professionals with corresponding employment contracts, where you are controlled by incentive agreements […]. And that includes money. Quite a bit, and accordingly you’re running on a wheel, so to speak.” [Participant #10, age 51–60 years, team lead]*.

Last, another participant stated that after observing low self-care at home office, switching back to company office was identified as a solution.

*“Well, I actually noticed that I have a very bad leadership style towards myself (laughs). Or rather, I don’t have any self-discipline. Meaning, in the first two months of the first lockdown, where I had to work in the home office, I didn’t really pay attention to myself. I started at eight o’clock, slowly got hungry at 12 o’clock, I’m just not much of a breakfast person, but then I noticed: a) there’s nothing at home, b) I can finish this e-mail and the call I just missed, I might as well call back and then another e-mail comes in and in the blink of an eye it’s 3 p.m. and I think to myself: ‘Okay, then you don’t need to prepare lunch anymore’, work anyway until 5 p.m. and only then start to take care of myself. Sometimes even longer. And I noticed that after two or three weeks. So that I was clearly more exhausted, I just […] had no daily routine. […] That’s why it was a good solution for me to go back to the office later on, where structures were a bit more clearly defined.” [Participant #8, age 31–40 years, team lead]*.

#### Comparison between leaders with and without pre-pandemic experience with virtual or hybrid teamwork

**Fig. 1:**
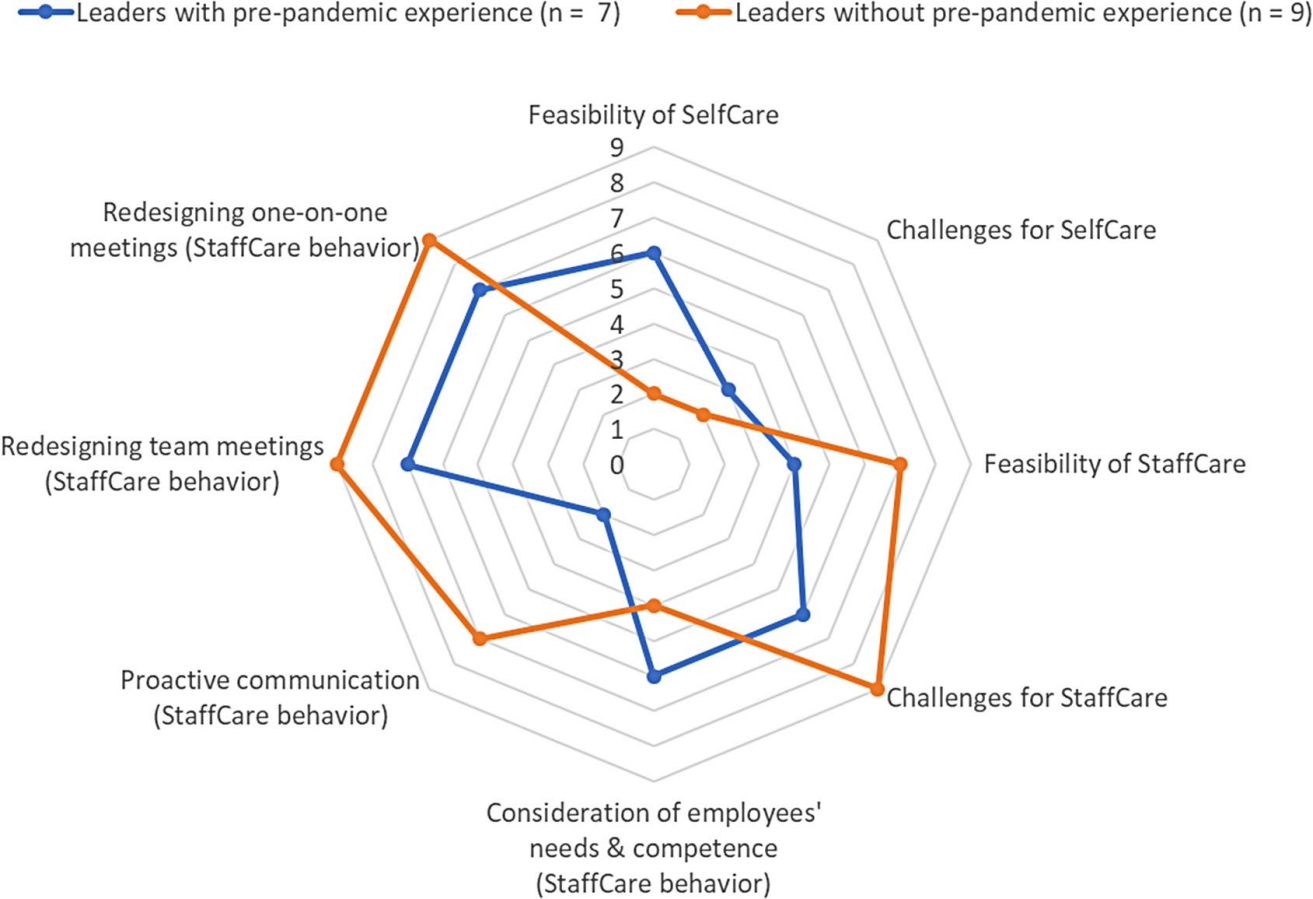
Explorative comparison regarding implementation of SelfCare and StaffCare in virtual teams during the COVID-19 pandemic. *Note* An explorative comparison was conducted between leaders with and without pre-pandemic experience with virtual or hybrid teamwork. The number on axes corresponds to the number of leaders who have named feasibility of SelfCare and StaffCare, associated challenges and respective leadership behaviors

A comparison between leaders with and without pre-pandemic experience with virtual or hybrid teamwork showed that almost all leaders with prior experience stated that SelfCare was also feasible for them in home office during the COVID-19 pandemic, although half of those participants also reported challenges in doing so. In contrast, two out of three leaders without pre-pandemic experience reported facing challenges and difficulties in implementing SelfCare after workplace changes due to the pandemic. However, one participant without pre-pandemic experience shared that practicing SelfCare was independent of work setting and that managing change was job-related and therefore familiar to him. Figure 1 presents the group comparison graphically.

*“I’m the head of change department, so change is something that I’m also kind of personally and professionally used to approach somehow.” [Participant #9, age 51–60 years, head of department]*.

### Implementation of StaffCare in virtual teams during COVID-19 pandemic

Overall, in terms of StaffCare in virtual teams during the COVID-19 pandemic, leaders reported on feasibility of implementation, but also on many challenges, and outlined their StaffCare behaviors in this context.

#### Feasibility of implementing StaffCare

With regard to StaffCare in virtual teamwork during the COVID-19 pandemic, a total of eleven leaders indicated that it was feasible for them to implement. Although many leaders emphasized that StaffCare implementation was feasible, but under more difficult conditions, the other half of those eleven leaders rated personal characteristics and behaviors of the leader (such as trust towards the team, openness, communication, and taking time) as more important for the implementation of StaffCare than actual differences between face-to-face and virtual collaboration.

*“So, I suppose - in the end - I’d say that it’s just as good or just as easy or just as hard as being present in the office environment. Because basically it’s about really listening to people. That is, to whatever concerns my colleague. And that’s maybe a little bit easier to read, you know, via nonverbal communication when I see the person, that’s for sure. But it’s actually more about, from my point of view, that the person is willing to be open.” [Participant #3, age 41–50 years, management]*.

#### Challenges for staffcare implementation

Although many leaders confirmed the feasibility of applying StaffCare in virtual teamwork, almost all participants described various challenges in implementing StaffCare in virtual teamwork during the COVID-19 pandemic: fewer cues in virtual communication, limited possibilities and quality of informal exchange, challenges maintaining or building proximity to employees as well as private challenges of employees (see Table 2).

**Table 2:** StaffCare in virtual teams during COVID-19 pandemic

Challenges for StaffCare implementation	StaffCare behaviors to cope with challenges
• more difficult perception of employees’ mental or physical health through digital communications due to fewer cues (12)	• consideration of employees’ needs and competence (e.g., by promoting autonomy, participation, reduction of hierarchies, communication of expectations regarding availability) (10)
• limited possibilities and quality of informal exchange via digital communications (4)	• proactive communication with employees whether within team or one-on-one (9)
• difficulties maintaining or building proximity with existing or new employees via digital media, in particular with a higher number of subordinate employees (5)	• redesigning team meetings (16):
	– *formal meetings*: increase in frequency of digital meetings (6), implementation of digital meeting etiquette (e.g., regarding timing, use of communication media) (8), enabling face-to-face team meetings (6)
	– *informal meetings*: organized rather than spontaneous contact with employees (3), additional meeting formats to encourage informal exchange within the team, e.g., regular digital team meetings, team events or lunch dates (9), encouraging informal exchange in formal meetings, e.g., via check-ins (5), enabling face-to-face meetings for informal exchange (3)
• private challenges of employees (e.g., due to childcare, limited housing, social isolation) (8)	• redesigning one-on-one meetings (16):
	– implementation of organized one-on-one meetings via digital media, by phone (11) or face-to-face (1) within varying, sometimes increased frequency (annually, every few months or weeks, weekly, in combination within professional exchanges, or on demand)
	– confidential conversations regarding current state of health and mind (10), work-related problems (2), to maintain contact (2)

*Note* The number in brackets corresponds to the number of leaders who have named respective leadership behaviors and challenges

#### Fewer cues in virtual communication

In total, twelve leaders reported difficulties in perceiving employees’ health status, feelings, or mood through digital communications – in comparison to face-to-face leadership before the pandemic. This was mainly attributed to fewer cues in virtual communication, especially when no video conference was possible and only voice was heard but no facial expressions or gestures were seen, also due to employees’ free choice to turn off video.

*“We can’t oblige anyone to turn on the camera, and there are colleagues who turn off the camera for a long time and don’t show themselves. And then, of course, it’s even more difficult when you only have the voice for a while.” [Participant #1, age 51–60 years, head of department]*.

The challenge was also described by some leaders by the fact that, despite a relationship of trust, most employees did not express problems to their leaders at an early stage.

*“That was one of my most difficult and biggest challenges. To pay attention to mental health. Because that’s even less possible, because the employees, I would argue we already have a great relationship of trust, and yet […] I don’t know everything. So, you can literally feel it. And then I just miss the facial expressions, the gestures, the body language, in order to really be able to better assess the situation.” [Participant #8, age 31–40 years, team lead]*.

In addition, it was shown that a more holistic perception of employees is limited not only in direct communication with the leader, but also in contact with other colleagues in virtual collaboration.

*“So basically, being able to lead well is of course also something that suffers, I think, because you obviously can observe employees less at work, for example. So, how does he deal with colleagues? You just don’t get to see that anymore. How does he interact with other departments? Usually, colleagues are here in the room and have a question, or other departments, and you can see directly: How does he solve it now? How does he deal with it? How does he react to stress? I don’t see all that anymore. I don’t see people talking to each other on the phone. That means I notice less and can therefore provide less information to them: What could you do differently? Instead, I usually only see the work output. So, what has the person worked on? How do I feel about that? And then, of course, you can do that digitally just as well as in the office. […] I believe that mere professional management, which is really about content, can also be done digitally.” [Participant #4, age 31–40 years, team lead]*.

Besides perception of employees’ mental health, perception of their physical health via digital communications was also found to be limited, although easier for prevention. Due to this limited perception, one leader pointed out that virtual teamwork requires employees to be more responsible for their own health than in the office.

*“I can’t assess whether employees in their home office are sitting and eating healthy and are taking breaks from work. So that, but each person is also responsible for his or her own actions and, as with everything else, I rely on my team. As a leader, I also see myself as an enabler. I provide everything that is possible for me and that is necessary […] And I’m only talking about physical health for now. It [StaffCare] is, well, it’s possible, but the employees’ self-responsibility is of course much more strongly required here than in the office.” [Participant #8, age 31–40 years, team lead]*.

In addition, two leaders stated that the more difficult perception of employees’ health status, feelings, or mood through digital communications was related to the fact that there was generally reduced communication that was more subject-related.

*“I (…) rarely get to call people and say: ‘Hey, I just wanted to chat’. Well, then one calls for a reason, and if one doesn’t have a reason, then one doesn’t call the people. Then one doesn’t know whether they’re doing well or badly.” [Participant #8, age 31–40 years, team lead]*.

#### Limited possibilities and quality of informal exchange

Overall, four leaders indicated that informal face-to-face contact with employees was much easier to implement before the pandemic. Due to the shift to digital communications as a result of the COVID-19 pandemic, informal contact was considered to be of limited possibilities and quality, e.g., due to insufficient technical equipment or less incentives.

*“I don’t think the informal aspect at the office can be replaced by a virtual coffee break. But it also has to do with the equipment. So, is it a virtual coffee break where people don’t see each other? Or do they have the resources to see each other via webcam? That has another quality. How many participants are there? That also plays a role. But basically, I would say it still can’t compensate for the classic face-to-face contact.” [Participant #9, age 41–50 years, head of department]*.

#### Challenges maintaining or building proximity to employees

Another challenge for five leaders was maintaining or building proximity to existing or new employees.

*“So, the biggest challenge for me is actually staying close, noticing when employees are not doing well and (…) taking the time to follow up. If you notice that something is wrong, you notice that relatively quickly in meetings, when it somehow becomes quieter around a person. But taking the time to follow up and ask questions is definitely a challenge, especially in this pandemic period, when we all tend to work more and are very busy with meetings. […] It’s good for me to have a lot of private conversations with the team, I would say, and to be close to them. But that’s also important to me as a leader. Maybe others are different in that respect. […] I think you have to know about each other, who you are and who you are in private, in order to really work well together. I think it’s extremely important to establish this proximity, especially when the team changes and new people join.” [Participant #5, age 31–40 years, team lead]*.

Especially in virtual collaboration, it was described as challenging to build personal relationships via digital media and not to lose employees over a long period of time – in particular with a higher number of subordinate employees.

*“Yes, the challenge is indeed to keep in contact, to develop a sensitivity for this. […] You have a completely different feeling about it, of course. There’s something wrong or the person is not doing well or whatever, and then you can work on it. That’s much more difficult now. So, without video anyway, but even with video. […] That means it’s more difficult to develop a feeling, a sense of being close to employees. And the more you have in direct reports, the more difficult it is to meet everyone’s needs. You have to be careful not to lose those who perhaps need to be addressed more. […] And then, of course, to what extent do you have insight into privacy?” [Participant #9, age 41–50 years, head of department]*.

One leader changed to a new company during the pandemic and reported on the challenge of establishing personal relationships via digital communications.

*“Well, I have one employee who is younger, but I am one of the youngest of them. We have many who are much older and have been with the company much longer, of course. And when is the right time to switch from “you” [formal, polite pronominal salutation in German] to “you” [informal, confidential pronominal salutation in German]? And I think […] in the non-digital world before Corona, there would have been several natural moments where you would have said, ‘Well, this is a good setting, now it’s time to do it’. But I haven’t found that here with my team yet.” [Participant #7, age 41–50 years, management]*.

#### Private challenges of employees

Last, eight leaders indicated that their employees’ private challenges during the COVID-19 pandemic had a negative impact on employees’ mental health and, consequently, posed a challenge to leadership. These ranged from the difficulty of combining childcare or homeschooling and home office, supporting one’s own parents, to limited housing (e.g., more difficult separation of work and private life in home office), social isolation, poor work-life balance, breakups, and financial problems.

*“But you just feel that there’s something and you also know that there are private problems, organizational problems, childcare, financial situation, a construction project that doesn’t work out, thousands of things, separation. And of course, all this has an impact on mental health. No one came out of the pandemic in a better mood.” [Participant #8, age 31–40 years, team lead]*.

#### StaffCare behaviors to cope with challenges

Reflecting on the challenges of StaffCare in virtual teamwork, leaders reported different leadership behaviors used to deal with them: consideration of employees’ needs and competence, proactive communication with employees as well as the redesign of team and one-on-one meetings (see Table 2).

#### Consideration of employees’ needs and competence

Overall, ten leaders acknowledged considering the needs and competence of their employees by e.g. promoting autonomy, participation and reduction of hierarchies. Some explained that cultural and organizational changes towards more self-responsibility, agility and eye-to-eye collaboration were implemented in their company.

*“I […] have been in leadership responsibility for […] more than 30 years. And things have changed in that respect, too. It used to be different. People used to lead in a different way. And now, it’s a change that I had to make and that I’m still involved in. But it’s a lot of fun. So, to call on employees to take responsibility for their own actions and to take them on board. […] Nevertheless, it is still disciplinary responsibility in a way that I also put myself in front of the group to provide support. I share responsibility for decisions, I stand in front of the group, and my colleagues also gave me feedback that my leadership behavior has changed in the last two years. […] Two years ago we had a restructuring and we are in the process of separating technical responsibility and disciplinary responsibility a bit.” [Participant #1, age 51–60 years, head of department]*.

One leader emphasized that in times of crisis, such as the COVID-19 pandemic, there is a need for leadership demanded by employees and requires clear and participative leadership behaviors.

*“This is what human history says, that especially in stressful situations, there is an immediate call for a leader. […] We no longer live in a time where typical hierarchical top-down decisions are made by one person and followed by others; instead, it’s about engagement and participative or coaching leadership. And that means somehow making sure that everyone is going in the same direction and, on the other hand, nurturing or enabling the greatest possible decision-making potential. And that’s how it is for us, that’s how I try to practice it. So, where it is possible or also wanted or just simply necessary, just to specify, to specify the direction, and on the other hand to enable the highest possible participation. […] During the weekend, I thought about this very carefully: Okay, what does that mean? And in the first few weeks, I led very closely, much, much more closely than I usually do. Because this was already somehow a crisis situation and, in a crisis, it simply helps people and they also call for […] ‘What does that mean now?’ and ‘Where are we going?’ and so on. And somehow, the clearer someone is, it’s like a ship is sinking and everyone looks to the captain and if he can calmly tell you: ‘Here are the boats and there’s the rope, please pull on it and everything will be fine and that’s where we’re going out now and that’s the direction where we’re going now,’ then it somehow goes well, I think, metaphorically speaking.” [Participant #6, age 51–60 years, head of department]*.

It was reported that individual needs of employees were considered and support was offered.

*“One of my very, very, very first trainings as a project manager, I learned to pay attention to three things when leading people. When distributing tasks, actually as a classic project manager: TPI. Task, Process, Individual. On the one hand, it’s about the task, what do you have to do? The other, process, how do we get there? And just as important, all equally weighted, individual, what does the person need to do this? Does she work at night? […] It doesn’t matter at all, but we have to combine these three things somehow, task, process, individual. And I think that’s quite important and helpful to remember that. And that’s always very easy to say, it’s also very easy for me to explain it that way, but to implement it, that’s the challenge. Because you have your own strengths, weaknesses and needs.” [Participant #7, age 41–50 years, management]*.

Furthermore, clear rules were established regarding availability and working hours, and expectations were communicated to support the boundary management between work and private life.

*“And we have clear rules, including absences. And also, my expectation is not to work in the evening or on the weekend, and I tell them that when that happens, and it does happen. Then someone calls me on vacation or on their vacation or on their day off. And then I’ll give them the hint right away, ‘Why are you calling me today? You’re off today.’ […] I practiced that [before the pandemic], too. But now I’m even stricter, I guess. Because that is more important to me. I notice by myself, this boundary management isn’t really easy, what concerns private and business, because it somehow becomes blurred. And I think you have to, I personally think it’s important to separate the two.” [Participant #10, age 51–60 years, team lead]*.

#### Proactive communication with employees

About nine leaders reported that in the wake of the COVID 19 pandemic, spontaneous informal exchanges with employees had to be replaced with proactive communications. This represented a major change for participants in which new strategies had to be considered and new behaviors learned in order to maintain rapport with each employee and within the team. Leaders described that proactively approaching employees was necessary, i.e., proactively asking questions, carefully listening, and taking more time for informal conversations. It was illustrated that more sensitivity was needed, especially with introverted employees who had become even more withdrawn as a result of virtual collaboration.

*“If I want to maintain contact, then I have to make sure that I do. So, if I don’t make sure that I have non-work-related contact with my colleagues and listen to how things are going, then nothing will happen, unless the other person is similarly active as I am […], but that was definitely a big change. Learning that you don’t just spontaneously meet a colleague in the hallway, but that you really have to make an active effort if you want to keep in touch. Especially with colleagues you don’t see at all because you don’t have anything to do with them professionally. Regarding your own team, I think you also need a different sensitivity than before, […] If you’re so far away and only meet someone in a team meeting and every two weeks in one-on-one meetings […], then I think you need even more sensitivity and in between you simply have to ask again: ‘Hey, is everything okay with you right now? Tell me again, how was your day yesterday? I remember you had an assessment center and you weren’t very satisfied.’ And I think you have to take more time, and that’s something I had to learn at the beginning.” [Participant #5, age 31–40 years, team lead]*.

#### Redesigning team meetings

All leaders described how they redesigned their team meetings for virtual collaboration during the COVID-19 pandemic. Several leaders emphasized that often this involved joint team deliberation and decision-making to address challenges.

First, some leaders outlined how formal team meetings were increased in frequency for virtual collaboration, as it was determined that there was an increased need for conversation. Accordingly, nine leaders reported that a new digital meeting etiquette was implemented. Thus, the timing of digital meetings was restructured. Due to an increased frequency of meetings in virtual collaboration, it was found that the timing of digital meetings was experienced as exhausting. Meeting duration in general, but also whole-day events, were shortened, for example, and meeting-free days were consciously scheduled.

*“We have introduced meeting etiquette. That means that no one is disturbed before eight o’clock and after seven o’clock. Except in management groups, where we have allowed it until 8 pm. After every 45 minutes, there’s a 15-minute break for digital meetings. There’s always an hour for lunch, and if a long meeting is scheduled for a whole day, it can’t go longer than four hours, because you’re simply more stressed digitally.” [Participant #12, age 41–50 years, head of department]*.

In addition, teams agreed on which communication media should be used for which purpose and how video conferencing systems should be used for verbal communication, e.g. camera on.

*“No, actually, in one of our first meetings as a team, where we kind of sat down together and discussed how we wanted to work in the future, especially under these conditions, we looked at which media and which channel we should use for which occasion. In other words, we tried to define […] when do I write an email and when do I post something in our Teams [a video conferencing system] channel, for example. […] But it took a while to get things going and, especially at the beginning, I got the feedback: “Oops, we haven’t done it like this for the last six months” and the last six months referred to the pandemic, to the beginning, where I was still on parental leave while my team was already there. And there was less of that, there was less communication simply through these channels. And because I have a great affinity for this, I simply pushed it a bit and used it more, and somehow it became second nature to everyone.” [Participant #5, age 31–40 years, team lead]*.

Face-to-face team meetings but also business trips were drastically reduced: some leaders reported regular (e.g., once per week) or infrequent (e.g., every three months) face-to-face meetings or hybrid team meetings at times, depending on changing policies during the COVID-19 pandemic. These were occasionally implemented for important meetings or to facilitate face-to-face exchanges and trust building.

*“I only travel to [large city in Germany] when I meet with a manager or have a very important appointment. I don’t think I’ve had any flights since March 2020, I haven’t been on a plane at all. And my business trips were maybe 20 to 30 in total, which is what I usually managed in a month.” [Participant #12, age 41–50 years, head of department]*.

Second, leaders described the redesign of informal team meetings. Overall, three leaders indicated that, compared to face-to-face leadership, informal contact had to be organized rather than emerging spontaneously, and new digital formats had to be considered.

*“And of course, you can discuss this much more easily over a cup of coffee than if you have to organize some kind of meeting for it.” [Participant #2, age 51–60 years, management]*.

Nine participants explained that they had implemented additional meeting formats to encourage informal exchange within the team: daily or weekly digital meetings (e.g., daily check-ins, virtual coffee breaks) for exchange and socializing, regular digital team events (e.g., game nights), joint digital sports sessions or healthy breaks, regular digital lunch dates, regular impulse weeks or webinars (e.g., on topics of collaboration and health-promoting work in the home office), as well as exchange between leaders was promoted via leadership workshops.

*“So, from my perspective, that totally helped to introduce these things like our daily or just a monthly team evening. We wouldn’t do a monthly team evening if it wasn’t for Corona [Covid-19 pandemic]. We never would have done that before. We used to meet maybe once every three months for a team evening. But we noticed that we were missing that and then we said: ‘Well, now it will be once a month and we’ll bake together, we’ll play virtual escape games together or everyone will bring a funny wallpaper for Teams [a video conferencing system] and we’ll guess where the person was or what the story is about this picture, just to create a bit of personal proximity and contact. And that definitely had a positive impact on our work.” [Participant #5, age 31–40 years, team lead]*.

Five leaders also referred to encouraging informal exchange in formal meetings, for example, by asking about current state of mind, e.g., during check-ins at the beginning of a meeting.

*“We start every meeting with a so-called check-in. It’s always about how we’re doing, and a few weeks ago there was a noticeable lockdown fatigue in Germany and, of course, in our team as well. And I addressed that too, and everyone just said they were sick of it and wanted to see people again and go out and so on.” [Participant #6, age 51–60 years, head of department]*.

One leader, on the other hand, also discussed the challenge of building trusting relationships only via digital meetings.

*“The second is the relationship level, which is actually difficult. Because part of that, especially in recent times, is that you go out to eat together as a team, in the evening as well, and in fact a certain degree of ease only sets in after a while. Not just after five minutes, but perhaps after you’ve been sitting together for two hours. And that is indeed a bit tricky, so I have tried to figure out how to create an online team event, for example, or to have a regular meeting every two weeks, and every two weeks I have another meeting with each team member, where the focus is not primarily on work content, but also on the other side of the business. And personally, I consider that a bit tough, because I don’t think we really get much out of it.” [Participant #7, age 41–50 years, management]*.

Last, three leaders indicated they had the opportunity to implement face-to-face meetings for informal exchanges, e.g., team events. One leader explained that he had learned during the pandemic that virtual collaboration in terms of content worked really well, and that social events should be deliberately planned for face-to-face meetings and separated from content collaboration.

*“What you can’t do [digitally] is to organize a […] social event. And we do that very consciously. […] And this separation is what I want to do now, I have to gather my experience and so far, it’s working very well. The employees are also on board and understand it. And they are now acting in the same way, separating content from personal events. […] And now that’s a reason to get together, saying, ‘So, we’re going to share,’ we’re looking forward to dinner, so it has become something special, and we’re also much more disciplined. When I decide: Now it’s about eating and exchange, […] then it’s simply more consequent. We didn’t manage that before. Most of the time, it was still about content-related topics at dinner.” [Participant #12, age 41–50 years, head of department]*.

#### Redesigning one-on-one meetings

Overall, all interviewed leaders reported organizing one-on-one meetings with their employees within varying frequency (annually, every few months or weeks, weekly, in combination within professional exchanges, or on demand). Most leaders described conducting these meetings via digital media or by phone. Thereby, some emphasized the challenge of not always being able to use the camera function or the need to implement personal exchanges in a more organized way.

*“So, you just have to exchange in a more planned way and take your time for that. In fact, I have consciously set digital meetings for this purpose, where we can then talk about it.” [Participant #15, age 31–40 years, team lead]*.

Only one leader stated implementing walks in the park for face-to-face communication for increased quality.

*“Whenever it was possible, we agreed that those things that are merely practical can also be done quite well online. But really chatting with someone, in quotation marks, is something we’ve often shifted to a walk over the last few months. So, when I’ve met with colleagues, I’ve rarely met to talk about something business-related. But more like: let’s meet in the park and go for a walk. […] Yes, and I think that has a completely different effect than to say: Okay, I’ll sit here online in front of the computer and we’ll talk for a while. So that’s already different.” [Participant #3, age 41–50 years, management]*.

However, two leaders reported implementing personal communication often within professional meetings, resulting in increased levels of communication during the pandemic. It was argued that this supposed relaxed conversational approach enabled many problems to be dealt with at an early stage.

*“I always started my meetings with personal topics, so that’s my conversation style. Always first: ‘How are you? Everything okay? I’m dealing with this and that, and what’s going on with you?’ […] and then, once it’s been discussed, I add: ‘Hey, I still have a subject-related issue’. Then it goes off. So that’s my way of managing the conversation. Optimally, I also finish with something personal, which has extended phone calls to an enormous length, but I’m firmly convinced that they were necessary. Because if I didn’t do that, I think the group would become […] more dissatisfied in the long run. And by always practicing this communication and not only preaching, but also doing it myself, I promote communication among each other. Often, really frequently, these supposedly relaxed conversations bring up things that are also professional. […] And I think I was able to eliminate a lot of trouble spots in advance, before they became major problems.” [Participant #8, age 31–40 years, team lead]*.

Regarding the purpose of holding one-on-one meetings, it was mostly stated that confidential questions were possible to ask about the current state of health and mind, e.g., about workload, stress perception, mental illnesses or particular private challenges during the pandemic. Within these conversations, leaders described how they sought individual solutions together with the respective employees, such as adjustments to work schedules.

*“Namely through personal conversations, through a lot of time and space. Some have already spoken up and expressed something like this, that it is somehow too much at the moment or this dissatisfaction in general, this insecurity. […] Sometimes it’s enough to somehow reduce the pressure a bit. There are different types of stress. I think some people just have a guilty feeling very quickly and are very conscious of their duties. And those are even the more difficult cases, according to my impression. And to say: ‘It’s okay if you don’t meet a deadline, as long as the company doesn’t collapse afterwards’. And to signal that: I’ve got my eye on you and I’ll defend you, if someone has a problem with this. So, there were situations like that.” [Participant #16, age 31–40 years, team lead]*.

In addition, individual participants stated that they hold confidential meetings in order to discuss work-related problems and to maintain contact with employees.

*“But I also see it as my job. So, I actually keep a list in which I write in: When was the last time you spoke to a colleague? And if that hasn’t happened for two months, I give them a call. So, I do try to keep in touch, because some people naturally drift away when you don’t get to see or hear them anymore.” [Participant #2, age 51–60 years, management]*.

#### Comparison between leaders with and without pre-pandemic experience with virtual or hybrid teamwork

A comparison between leaders with and without pre-pandemic experience with virtual or hybrid teamwork showed that seven leaders with no prior experience reported being able to implement StaffCare in virtual teamwork during the COVID-19 pandemic, compared to four leaders with prior experience. In both groups, about half emphasized that StaffCare implementation was carried out under more difficult conditions. Almost all leaders described various challenges in implementing StaffCare in virtual teamwork during the COVID-19 pandemic and both groups differed little in terms of what they said about the challenges: fewer cues in virtual communication, limited possibilities and quality of informal exchange, challenges maintaining proximity to employees as well as private challenges of employees. Last, the group comparison regarding StaffCare behaviors revealed that all leaders, regardless of prior experience, reported having adjusted team and one-on-one meetings. However, the majority of leaders with no prior experience more often stated using proactive communication with employees, compared to only two leaders with prior experience. Likewise, almost all leaders with prior experience reported considering employees’ needs and competencies, compared to only four leaders without prior experience (see Fig. 1).

### Preconditions of HoL in virtual teams

Leaders named several personal, organizational, social, and technical preconditions as conducive to the use of HoL in virtual teams (see Table 3).

**Table 3:** Preconditions of HoL in virtual teams

Preconditions	Details
Personal preconditions (15)	• characteristics of the leader, e.g. empathy, trust (12)
	• self-care of the leader (9)
	• leader as role model (7)
	• leadership experience (2)
Organizational preconditions (16)	• supportive management (10)
	• supportive, open-minded corporate culture (15)
	• health-promoting working conditions (10)
	• wide range of institutional services (13)
	• employee-oriented, supportive work council (3)
Social preconditions (13)	• social support by team, e.g. through employee ownership (12)
	• social support by colleagues or own supervisor (4)
Technical preconditions (5)	• stable internet connectivity (2)
	• sufficient technical equipment for all employees (4)
	• IT support by organization (1)

*Note* The number in brackets corresponds to the number of leaders who have named respective preconditions

#### Personal preconditions

Almost all leaders stated that leaders’ specific characteristics and behaviors, self-care, role modeling, and leadership experience are basic preconditions for practicing HoL in virtual teams.

About twelve leaders discussed key characteristics as important preconditions for practicing HoL. Thus, seven of these participants reported that leaders need empathy in order to be able to perceive moods and communicate sensitively, even at a distance and via digital communication. Another seven participants stated that trust is essential, especially to avoid micromanagement in virtual collaboration, and can be built through sincerity, authenticity, transparency, and commitment. Additionally, it was described that leaders should be open (both to employees and in terms of how they work), have a genuine interest in their employees, and have awareness and sensitivity to issues around health.

*“In any case, you first have to be empathetic, because otherwise you’re probably not interested in whether you’re helping the other person to develop, whether you’re helping the other person to cope with such a situation. And you have to be quite sensitive or attentive in order to notice that. It’s just something different than in the office. Even there, I would argue, there are people who wouldn’t notice or wouldn’t question it. And you have to be critical accordingly, i.e., you have to question whether the employee dares to say that or whether he or she doesn’t want to admit it.” [Participant #4, age 31–40 years, team lead]*.

For this, one leader explained that the leadership task should be considered relevant and time should be devoted to it.

*“The freedom of leadership or the leader to say that these topics, these team meetings and also these one-on-ones, that they are important and that time is also allocated for them. I think leadership is often seen a bit like that: Yes, you still have a team here, in addition to your actual job. And I think that’s often somewhat difficult for many people to understand. How much time do I really have to spend on my team in addition to my actual job?” [Participant #16, age 31–40 years, team lead]*.

As a related point, about nine leaders explained that their own SelfCare, the health-oriented way they treat themselves, is relevant to practicing StaffCare. They described that in order to achieve this, first, one’s own health and well-being should be considered important; second, awareness should be practiced (e.g., via self-reflection, a positive self-perception, personal attitudes, and knowledge of health-promoting work); and third, health-promoting behaviors should be practiced (e.g., exercise, spiritual rebalancing, spending time in nature, switching off from work, setting boundaries).

*“Yes, of course you have to prioritize this issue for yourself, otherwise I think it’s difficult to implement it well for others. If you didn’t care at all about your own health and well-being, I can hardly imagine that I would be able to do this well for others.” [Participant #9, age 51–60 years, head of department]*.

Four leaders emphasized that high job demands, such as intense deadline pressure or high workloads, or unfavorable personal conditions, such as housing conditions, may cause increased stress and impede one’s own self-care.

*“That also depends on my personal mindset and my constitution at that moment, for sure. I noticed that my daily state, in quotation marks, varies a lot. There are days when I have really good sensors for it and can react well to it. And on other days where I’m just so caught up in my to-dos that I somehow don’t have an eye for it. […] Or I notice it the next day that something happened. But yes, it also depends totally on how I’m feeling myself and I had to learn to take care of my health first, in order to be able to help others from a strengthened position. […] These are exactly the days when I don’t have a free minute and meetings follow meetings.” [Participant #5, age 31–40 years, team lead]*.

Thereby, one person explained the importance of continuously applying health-promoting lifestyle patterns in order to be able to recall them more easily in stressful situations.

*“If I’m not used to balancing myself out in sports or spiritually, if I’m basically rather negative or have difficulty dealing with changes or pay little attention to myself, have a poor relationship to myself […]. And this is then multiplied by stressful situations or by a pandemic, I think, unfortunately. […] There is just a special situation in a pandemic and everything that was there before in terms of good habits and life coping strategies can be accessed more easily.” [Participant #6, age 51–60 years, head of department]*.

Furthermore, seven leaders stated that the leader has a duty of care and an important role model function (among other things, for SelfCare, trusting and fun cooperation, as well as proximity, openness and transparent communication).

*“Of course, the role model function is very important. As a leader, I have to be healthy myself and have an eye for what that means, what’s good for me and what’s not good for me. I have to know what I shouldn’t do and how I can set an example. For example: I will, and there I am also quite clear, I will always take my vacation leave and I will not take my 30 days over too. I don’t have problems like that, because I know for sure that it’s extremely important for me to get this time off and just get out and relax on vacation. That is super important to me. Just as a small example. And I will always pass that on to my employees: ‘Do that.’ I will also set an example, because I think it’s extremely important that not everything is about work 365 days a year. That means the role model function is very important, so I believe that as a leader I must have understood what that means and must behave accordingly. Otherwise, I can’t expect my employees to behave healthily themselves. That is also the case with various other issues.” [Participant #15, age 31–40 years, team lead]*.

Last, two leaders added that leadership experience, e.g., via intensive leadership training, is conducive to the implementation of HoL.

*“However, I also had 15 years of leadership experience at the [name of the company] and there were always very intensive training programs for leaders. How to deal with addictions, so I’m really very sensitized. I’m also very aware of what signals to look at. As a young leader, you are often overwhelmed: ‘Oh, you can’t talk about that now, if he always smells like alcohol.’ You have to find ways to deal with that somehow. But you learn to pay attention to that: When does something change? Or when does an employee’s behavior change when he becomes more and more quiet? And then you must also have the courage to ask: ‘Are the tasks inappropriate? Or are you feeling bad? Can we do something?‘” [Participant #14, age ≥ 61 years, team lead]*.

#### Organizational preconditions

All leaders indicated organizational preconditions for using HoL in virtual teams: from a supportive management, to a supportive, open-minded corporate culture, health-promoting working conditions, a wide range of institutional services and to an employee-oriented, supportive work council.

First, a total of ten leaders argued that management takes on a supporting role when it recognizes the relevance of occupational health and anchors it at a structural level, as well as acting as a role model.

*“Yes, so our divisional management, with whom I have also worked closely in recent months, it already helps if they also keep an eye on such issues and also clearly communicate to employees that we should pay attention to each other, that if we are not doing well, we should talk openly about it with our supervisor or with other colleagues and that we need to actively communicate. Such calls came more frequently in recent months because this is already an issue and we simply see, especially in the HR department, that employees also face this issue of working so much from home and being in the office so little. And we try to balance that out as much as possible. But it really helps when management emphasizes this again. Every four weeks, there’s a letter from the management, another official one, in addition to various other formats, where it’s reminded that everyone should take the time to do this. And of course, that helps me as a leader too, in that it gives us even more legitimacy to ask.” [Participant #5, age 31–40 years, team lead]*.

Second, fifteen leaders reported that a supportive corporate culture is another precondition for the implementation of HoL. They described that an open-minded corporate culture is beneficial for employees to discuss problems.

*“And I would say that this openness, which is needed, is much more important than whether I meet the person face-to-face on site or not.” [Participant #3, age 41–50 years, management]*.

Furthermore, several leaders explained that a cultural change in their company towards more self-organization or agile collaboration meant better conditions for leadership.

*“Our management board or advisory board has just launched a project, or is in the process of launching one, where the topic is: culture. Culture in our company. It has been recognized that we need to implement a cultural change so that we can work together successfully in the future. And we are currently in the process of doing so. As I said, we started two years ago with an agile mindset, with agile methods. […] The management board and so on are also for now mostly on a first-name basis. Everyone is addressed by their first name. That wasn’t the case with us in the past. […] And the communication channels, open communication and transparent communication. The fact that the management board now also publishes objectives, their own objectives and strategy, and so on, in teams. That was a bit different before. That was always hierarchical. The management board passed this on to its department heads, and they perhaps passed it on to the team leaders. And now it is passed on directly from management to all employees. So, the type of information policy has already changed.” [Participant #1, age 51–60 years, head of department]*.

In this regard, three participants also explained that there were still challenges in adapting to new working conditions during the COVID-19 pandemic, e.g., due to a change-resistant corporate culture or during cultural change.

*“One thing is a lack of experience with introducing and implementing change. The organization is not used to this, and perhaps it is not wanted, because it is also somewhat uncomfortable.” [Participant #7, age 41–50 years, management]*.

Yet two leaders explained that the corporate culture may differ between teams and that good teamwork depends more on leadership and team culture than on corporate culture.

*“I believe that this always depends on the team. I think you can be in a company that is super open and super cool and new work and so on, but you can have a manager who is just in the numbers and who will miss all empathy and then not even see that other employees just don’t find it that cool. And conversely, you can have a company that has been on the market for a relatively long time, perhaps a bit encrusted in some way, which is probably the case with most long-established companies […]. And you can just as well have teams that are just totally cool and agile and strong in sharing. That’s more how I would put it.” [Participant #16, age 31–40 years, team lead]*.

Third, ten leaders suggested that working conditions of employees and leaders in the company can influence leadership. They explained that high workloads, a lack of meaningfulness in the job, an inadequate workplace environment and ergonomics in the office or home office, or non-flexible working hours can impair HoL.

*“Of course, we have flexible working hours, which certainly adds to that. Especially in the home office, it’s possible at any time […]. So, for example, if an employee works for two hours, rides his bike for an hour, takes a shower and then works again for three and a half hours, then in case of doubt goes out with the dog and then works again for three and a half hours, as an example. So, the flexible work schedule is, of course, very convenient at that point. That’s certainly helpful for healthy leadership.” [Participant #9, age 51–60 years, head of department]*.

Fourth, thirteen leaders added that a wide range of institutional services in their companies was perceived supportive for implementing HoL: i.e., a wide range of professional training for leaders and employees, workplace health promotion offers (such as healthy breaks, guided walks, sports and diet offers), regular employee surveys and consequent deduction of actions, communication opportunities with management (such as open-door meetings or digital events), networking offers and monetary offers (such as special leave days or a health budget for every employee).

*“So, I have to say, I’m totally happy with my position and with my opportunities right now. And, as I said earlier, if the employee wants to, we can discuss everything. […] So I can actually offer my employees everything they need so that they can also work in a healthy way.” [Participant #1, age 51–60 years, head of department]*.

Last, three leaders argued that an employee-oriented work council is a supportive precondition for implementing HoL, especially in times of changing working conditions. It was described that a restrictive work council hindered the introduction of digital media or flexible working hours.

*“The work council sometimes (laughs) has ideas that are not necessarily good for all employees. Because the work council sometimes wants to protect employees, but, for example, what I just said, if someone prefers to work in the evening because the kids are in bed at eight and then he can work well again, but the work council insists on saying: ‘Okay, only until 8 p.m., you’re not allowed to work any longer.’ This would be counterproductive for some employees.” [Participant #1, age 51–60 years, head of department]*.

#### Social preconditions

Overall, thirteen leaders stated that support by their team, by other colleagues or own supervisors are further conducive preconditions for implementing HoL in virtual teamwork.

First, a total of twelve leaders indicated that employee characteristics such as openness, trust, honesty, engagement, and ownership were experienced as supportive. Thereby, leaders explained that the implementation of HoL also requires the competence and trust of employees to open up in collaboration, to communicate honestly and to engage in this type of leadership, e.g., via mutual care for one another.

*“And that the person, the employee, the colleague, that they participate. That they also open up. So, as I said, I can only talk to someone openly and honestly, I would say, if they are also open and honest. Otherwise, I may be on the wrong track and do him no good at all, because he may not give any or not the right feedback or honest feedback. For me, that is always an interplay.” [Participant #1, age 51–60 years, head of department]*.

In addition, leaders described that employees’ individual responsibility regarding their physical and mental health is challenged more in virtual than in face-to-face collaboration.

*“Yes, that’s exactly where it gets difficult, because the fact that you don’t see people in person means that everyone works with an incredibly high level of self-discipline. In this case, self-discipline doesn’t mean working as long as possible and as much as possible, but knowing where the limits are, where you stop. It may well be the case that someone demands more and more and at some point, you have to say, ‘Stop. Here’s a limit right now.‘” [Participant #11, age 51–60 years, head of department]*.

According to one of those leaders, knowledge of team members through earlier face-to-face collaboration was conducive to virtual collaboration.

*“One influence is certainly how well I know my team, my employees, the environment. I can imagine it being extremely difficult when, as a new leader, you start working for a company on a fully digital basis and have perhaps never seen your colleagues or employees before, or have only seen them digitally. So that is a framework condition that I imagine to be very difficult. […] So given the precondition of saying: […] I know the employees, I know what they can do, what they can achieve. Strengths and weaknesses. You can say that this also works, even digitally to a certain extent. But in the other situation, I think it’s much more difficult to first earn this trust.” [Participant #14, age ≥ 61 years, team lead]*.

Second, four leaders added that colleagues (e.g., for exchange on leadership topics) or own supervisors (e.g., no performance pressure, open-mindedness, autonomy) were experienced as supportive for practicing HoL.

*“Yes, colleagues. Because I’ve been with the company for so long, I’m relatively well connected and have one, two, three, four contacts or colleagues who can help me with every topic or question. In this respect, I think I’m also quite well supported. Even beyond the site, i.e. nationwide, yes.” [Participant #9, age 51–60 years, head of department]*.

*“Yes, my supervisor. He allows me to lead in this way. He doesn’t expect me to deliver fixed numbers that have to be achieved, no matter what. He is open for ideas, for new approaches, for suggestions. He also helps me to keep negative influences away from the team and to protect the group. So, I can only say that my kind of leadership would not be possible without my boss.” [Participant #8, age 31–40 years, team lead]*.

#### Technical preconditions

Overall, five leaders indicated that a stable internet connectivity, sufficient technical equipment for all employees and IT support by the organization are among the preconditions for implementing HoL in virtual teamwork.

*“If there were any problems, we have a good IT department, which serves as a direct point of contact.” [Participant #4, age 31–40 years, team lead]*.

On the one hand, some leaders indicated that their company had fully met technical preconditions, while still others indicated that challenges still existed, e.g., no uniform technical equipment for all employees, no standardization of digital communication channels and server infrastructure or a stable internet connectivity during international virtual collaboration.

*“For people who live in regions or countries where the internet connectivity is not quite as reliable, it can happen that when they switch on their video stream, people suddenly become hard to understand or disappear from their meeting completely. So, they usually have to dial in acoustically or even use the telephone.” [Participant #11, age 51–60 years, head of department]*.

#### Comparison between leaders with and without pre-pandemic experience with virtual or hybrid teamwork

**Fig. 2:**
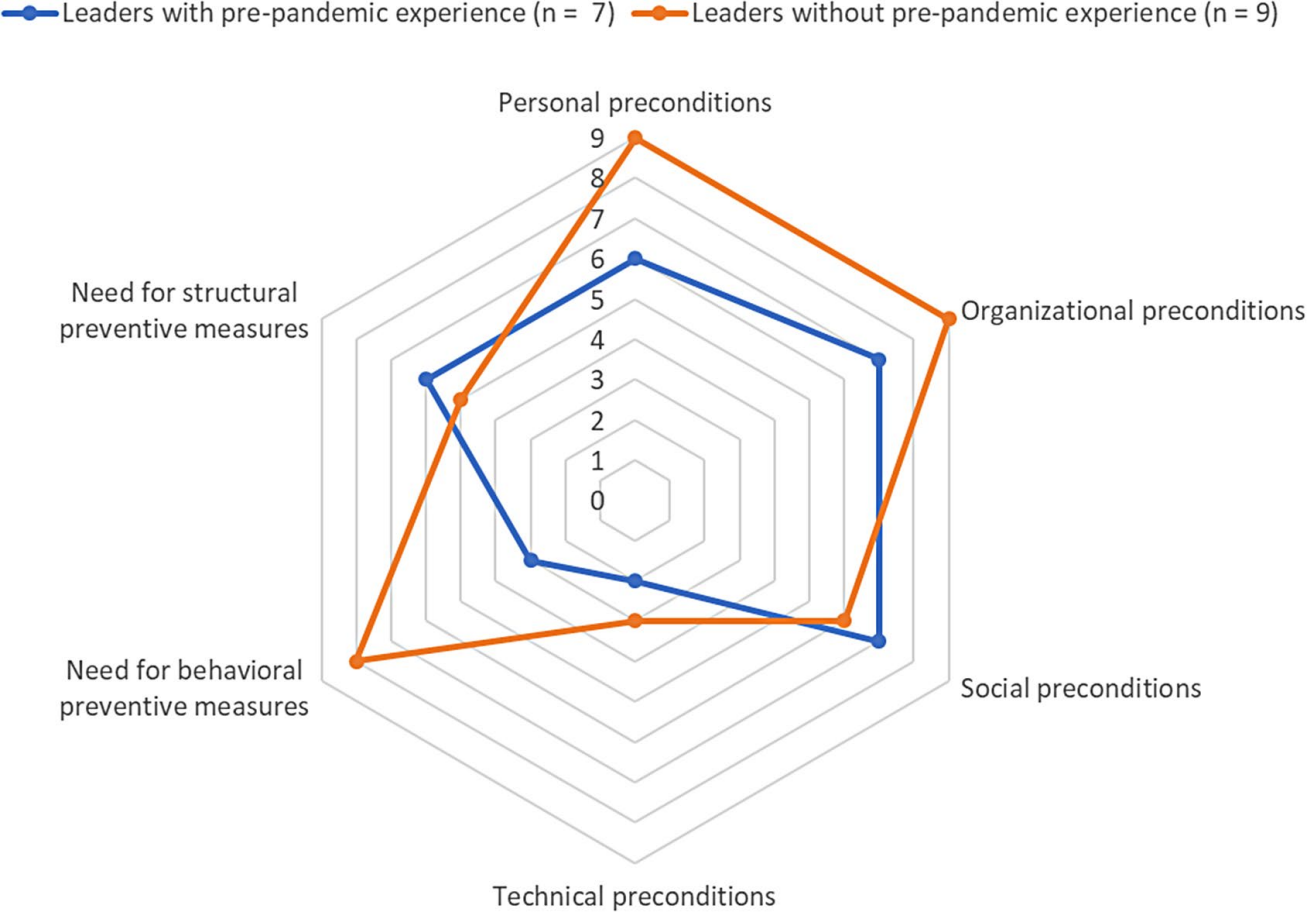
Explorative comparison regarding preconditions and preventive measures for promoting HoL in virtual teams. *Note* An explorative comparison was conducted between leaders with and without pre-pandemic experience with virtual or hybrid teamwork. The number on axes corresponds to the number of leaders who have named respective preconditions and preventive measures for promoting HoL in virtual teams

A comparison between leaders with and without pre-pandemic experience with virtual or hybrid teamwork showed that almost all leaders reported personal, organizational, or social preconditions for HoL in virtual teams. In contrast, technical preconditions were reported less frequently. With regard to social preconditions, it was found that all leaders with prior experience perceived their team as supportive, in contrast to half of leaders without prior experience. Figure 2 displays the group comparison graphically.

### Preventive measures for promoting HoL in virtual teams

All leaders named existing or needed behavioral and structural preventive measures - which they found or would find helpful - to promote HoL in virtual teamwork (see Table 4).

**Table 4:** Summary of preventive measures for promoting HoL in virtual teams

Behavioral preventive measures	Structural preventive measures
**Further training for leaders (8):**	**Support from management (5):**
• e.g. on health-oriented, virtual or hybrid leadership • best practice exchange programs • coaching	• communication of appreciation • transparent information flow • support for cultural change towards enhanced autonomy • long-term, preventive focus on health and well-being of the workforce
Further training for all employees (5): • e.g. on self-care • more incentives • follow-up regularity	Improvement of technical equipment and general digitization processes (7): • uniform equipment of all employees with necessary technology such as webcams • lean communication channels • new technical solutions for digital, remote team and department development • further digitization, e.g. server, processes

*Note* The number in brackets corresponds to the number of leaders who have named respective preventive measures

#### Behavioral preventive measures

About eight leaders discussed on behavioral preventive measures that are beneficial for leaders themselves: further training on health-oriented, virtual or hybrid leadership, best practice exchange programs or individual coaching offers. Six of them referred to existing offers in their company.

*“Yes, I would really like to see training on how to manage hybrid employees well, because that will be the next challenge. How can I integrate employees from the home office as well as those who are in the office? We will probably end up with a hybrid version, which is why I think it makes totally sense for us to take a closer look at what that means for us in concrete terms.” [Participant #5, age 31–40 years, team lead]*.

In addition, five leaders outlined that behavioral preventive measures for all employees would be beneficial for developing employees’ self-care, such as creating more incentives to increase awareness for health promotion. Two of them referred to existing offers in their company.

*“Yes, self-responsibility, self-discipline, self-care. So that’s what I’m missing and that’s what I know one or the other employee was missing, also in the course of ‘Now I have to take care of my child and then I have to keep track of my working hours and then there’s another person calling me’ and that you don’t take yourself as important anymore. So, I think that if there were offers to heed alarm signals, […] this issue - self-care - is quite present at the moment anyway.” [Participant #8, age 31–40 years, team lead]*.

Thereby, one participant pointed out that rather mandatory and especially regular offers for all employees are needed to promote the implementation of HoL.

*“I think there are enough offers, I think one should achieve a certain regularity, sensitize again and again. […] This is nothing new, but what you notice in daily business is that it gradually gets lost again and again. So, if you are not very disciplined or if you don’t get an impulse again, then it is increasingly pushed back to the background. […] That’s why I think it would be important and good not only to make offers, but also to simply introduce mandatory seminars in this area.” [Participant #9, age 51–60 years, head of department]*.

In total, four leaders expressed no further need for behavioral preventive measures as there was already a sufficiently large range of services in their company.

*“So, I’m not missing anything. I hope that there won’t be any more, because then something completely different will happen, then we’ll have an overflow of communication.” [Participant #11, age 51–60 years, head of department]*.

Another participant highlighted the need for both behavioral and structural preventive measures.

*“I believe that in case of doubt, both are good. So, it basically goes hand in hand.” [Participant #3, age 41–50 years, management]*.

#### Structural preventive measures

About five leaders discussed support from management as beneficial for practicing HoL. This included appreciative and transparent communication to employees, especially during times like COVID-19 pandemic, when a great deal of uncertainty and frustration was prevalent among workforce.

*“And what is left behind […] I think it has a lot to do with appreciation. […] that comes first of all from the leader, from the direct one. But I think above all, when the organization becomes larger, then at some point it stops. And at some point, employees start to think that they [management] are only interested in whether the business is running well. […] Even if they [management] have good intentions, many decisions sometimes come across differently to the broad group of employees. And I think that was one issue. At a time when there is a lot of frustration, combating this frustration efficiently has to be managed by the leader. They have to be given the tools, because if they don’t, the same thing will happen that takes place in the office. This, what do you call it? Trash talk? (laughs). That people vent their frustration in an unhealthy way. […] It [affects communication] from the very top down to the lower leadership levels, where communication needs to be good as well.” [Participant #16, age 31–40 years, team lead]*.

Leaders explained that hierarchical management is a barrier to the practice of HoL. Management was also called upon to support staff during cultural changes towards enhanced autonomy by providing guidance. In this context, one leader explained that a long-term, preventive focus on health and well-being of the workforce is required from management.

*“Well, I think it’s very important that even in a medium-sized company like ours, the focus is on [health]. In order to prevent people from getting ill in the long term or quitting or simply not being able to perform at the level they normally would. And I know that from other companies as well. That’s just something, yes, there’s no focus on it. There is only a focus when you suddenly have a wave of employees quitting or poor quality, so you think: My gosh, what’s the reason for that (laughs)? And then to recognize that some colleagues are just completely overworked or underworked, that usually takes a while. Because they run with the team and before you realize that something is wrong, it’s often too late.” [Participant #14, age ≥ 61 years, team lead]*.

Furthermore, seven leaders emphasized the need to improve technical equipment and general digitization processes, e.g., uniform hardware and software equipment for all employees with necessary technology such as webcams, lean communication channels, more stable servers or new technical solutions for digital, remote team and department development.

*“Enabling even more digitization, perhaps having fewer channels at the same time. The fact that we have both Skype and Teams [two video conferencing systems] at the same time is a bit annoying, to be honest. And I would like to see greater speed in the process of migrating. Skype is supposed to be shut down and we’re supposed to use Teams for everything, but that won’t be implemented until the end of the year.” [Participant #5, age 31–40 years, team lead]*.

#### Comparison between leaders with and without pre-pandemic experience with virtual or hybrid teamwork

A comparison between leaders with and without pre-pandemic experience with virtual or hybrid teamwork showed that almost all leaders with prior experience identified a need for structural preventive measures. Regarding behavioral prevention measures, four leaders expressed no further need, referring to existing services offered by their company that they found helpful. In contrast, almost all leaders without pre-pandemic experience in virtual or hybrid teamwork reported a need for behavioral preventive measures. Moreover, five leaders indicated a need for structural prevention measures (see Fig. 2).

## Discussion

The aim of the present study was to conduct qualitative interviews with leaders in order to gain new insights into the experiences and challenges for (ad hoc) SelfCare and StaffCare in virtual teams in times of the COVID-19 pandemic, into preconditions that may enable HoL in virtual teams and into preventive measures that may promote the use of HoL in virtual teams. To our knowledge, this study is the first to provide empirical results on preconditions of HoL in virtual teams during the COVID-19 pandemic and on explorative, qualitative group comparisons between leaders with and without pre-pandemic experience with virtual or hybrid teamwork. Reflecting upon our theoretical framework, the HoL model [16], an extension of the model by a holistic approach to context should be discussed (see chapter on implications for future research).

### Implementation of SelfCare in home office during COVID-19 pandemic

Similar to present study results, previous studies indicated that leaders of virtual or hybrid teams also faced increased job demands and challenges during the COVID-19 pandemic, such as additional crisis management tasks [7], longer working hours due to increased workload [8], impaired mental health [9], and increased exhaustion with high appraised e-mail overload [6]. In times of increased job demands, higher levels of SelfCare may be beneficial in order to be able to take care of one’s own health [18]. In relation to our research question of what experiences and challenges virtual leaders faced regarding (ad hoc) SelfCare during the COVID-19 pandemic, currently, only two studies exist that have examined SelfCare of leaders working in virtual teams. Thus, a quantitative study with leaders who led remotely from home offices during COVID-19 pandemic showed that leader SelfCare was effective in home office setting and had a positive impact on, amongst others, leader’s health, well-being, job satisfaction, engagement, and commitment [9]. Likewise, present qualitative results indicated that leaders who experienced no challenges in implementing SelfCare in their home offices expressed job satisfaction with their flexible and self-responsible working conditions. Another qualitative interview study with virtual team leaders prior to COVID-19 pandemic demonstrated that SelfCare can be applied in virtual teams as well. In this study, leaders reported a high relevance for their own health, a high awareness of changes related to their health, and specific SelfCare behaviors: physical activity, boundary management, healthy nutrition, sleep, recreational activities, social exchange, time management, use of occupational health services and mental handling of stress [22]. Similarly, the leaders of the present study also reported behaviors such as self-reflection and self-discipline to apply SelfCare. In contrast, however, our study presented challenges that were associated with the implementation of SelfCare during the COVID-19 pandemic, particularly among leaders who had no pre-pandemic experience with virtual or hybrid teamwork. Overall, no other comparable studies exist that analyzed leaders’ self-directed leadership by their pre-pandemic experience with remote, digital collaboration.

### Implementation of StaffCare in virtual teams during COVID-19 pandemic

Referring to our research question, regarding the experiences and challenges of virtual leaders in terms of (ad hoc) StaffCare during the COVID-19 pandemic, the current state of research on the feasibility of implementing StaffCare, associated challenges and StaffCare behavior is discussed as follows. In terms of subjective perceptions on feasibility of implementing StaffCare in virtual teams, there exists only one other qualitative study with virtual leaders, but from the period just before the COVID-19 pandemic [22]. In comparison to the present study, this previous study showed that all interviewed virtual leaders rated the relevance of employee health as high as their own and the majority stated that they were able to perceive changes in the health status of their employees also in the virtual work context. The sample consisted of leaders who were familiar with virtual leadership and collaboration – unlike the leaders in the present study, half of whom had to make an ad hoc switch to virtual collaboration [22]. Accordingly, results of the previous study are rather comparable to leaders with prior experience from the present results.

Similarly, to how the majority of leaders surveyed in this study mentioned challenges in perceiving employees’ mental or physical health through digital communication due to fewer cues, previous studies also demonstrated leaders’ challenges in implementing StaffCare. For example, a quantitative study surveying over 1,300 leaders shed light on leadership-specific challenges in the home office during the COVID-19 pandemic, with over 45.7% of leaders reporting that it was difficult to notice when someone needed support and over 41.8% stating that it was difficult to keep track of how much their own employees were actually working [9]. Three qualitative studies with leaders during the COVID-19 pandemic discussed that the transition to home office created difficulties in perceiving their employees (e.g., obtaining a sense of how their employees were managing with their tasks, detecting employees’ individual level of motivation or identifying employees’ support needs), which was experienced easier in the office before [7, 8, 31]. Another qualitative study with virtual leaders before the COVID-19 pandemic revealed that physical distance and reduced communication quality diminished leaders’ awareness of employee health status [22]. And yet, a review on leadership when working from home also referenced several studies that outlined the challenges for leaders to perceive subtle signals or non-verbal communication via digital technologies or online meetings [47]. Independent of remote setting, an experimental vignette study showed that leaders’ awareness of warning signals (for early detection of emerging depression and burnout among followers) may be facilitated by clarity of displayed warning signals of employees. In this study, leaders’ awareness was highest when they perceived a combination of warning signals: employees’ poor performance and socioemotional withdrawal [48]. Furthermore, our study found limited possibilities and quality of informal exchange via digital communications to be challenges of leaders in implementing StaffCare. Similarly, a quantitative study by Krick and colleagues [9] on HoL in home office during COVID-19 pandemic illustrated that as digital communication increased, less informal communication occurred and that 37.7% of leaders reported that it was difficult to exchange ideas spontaneously with employees. Also, a recent quantitative survey by Fraunhofer Institute for Industrial Engineering IAO found that about 35.1% agreed that networking among colleagues had worsened and 23.2% perceived a reduced interest in social exchange and related company offers due to working from home [49]. Other qualitative studies on HoL and transformational leadership during COVID-19 pandemic identified a lack of social presence, limited options for informal conversations, communication difficulties, lack of mutual trust and employees’ lower willingness to talk about problems when using electronic forms of communication as challenges for leadership in a remote setting [7, 23]. In line with our results on leaders’ difficulties maintaining or building proximity with existing or new employees via digital media, previous studies also demonstrated more intensive relationship design and maintenance as a challenge of virtual leaders [50–53]. Thus, numerous studies on virtual leadership highlighted the relevance of trust building for successful collaboration [22, 54, 55]. Last, our study indicated that leaders perceived their leadership to be impacted by employees’ private challenges during the COVID-19 pandemic (e.g., due to childcare, limited housing, social isolation). Likewise, a panel survey in Germany during COVID-19 pandemic indicated an increase in gender and socioeconomic inequalities in parents’ psychological well-being, such as higher parenting stress among parents working from home and parents with financial insecurity [56]. Further, a review demonstrated that also personal factors, such as living conditions or perceptions of isolation or loneliness, impacted employees’ mental health [57] and their perceptions of remote or home office work [5]. Given the higher risk of experiencing isolation in virtual teamwork, pre-pandemic studies pointed to a potential mitigating effect of leadership on perceptions of social or professional isolation among digitally collaborating personnel [11].

Moreover, our study discussed StaffCare behaviors of leaders dealing with particular leadership challenges during the COVID-19 pandemic. These included, consideration of employees’ needs and competences (e.g., by promoting autonomy, participation and supporting boundary management), replacing spontaneous informal exchanges with proactive, planned communications in virtual collaboration, as well as restructuring their formal as well as informal team and one-to-one meetings. In comparison to our results, a quantitative study on digital StaffCare in home offices during the COVID-19 pandemic illustrated that there were great differences in leaders’ self-reports and employees’ peer-reports of health-promoting leadership behaviors [9]. Another qualitative study before the COVID-19 pandemic on StaffCare in virtual teams found that leaders reported trust building activities, health-oriented communication, support in boundary management, implementation of face-to-face meetings, and delegation of decision-making authority and responsibility as relevant leadership behaviors in this context [22]. Further, a review on leadership when working from home identified six overarching leadership behaviors that were beneficial for employee well-being and work performance: communicating and informing (e.g., frequent and regular communication), controlling and setting boundaries (e.g., transparent guidelines regarding working hours and breaks, availability requirements, supporting employees with boundary setting), allowing autonomy (e.g., showing trust in employees’ performance and responsibility, delegating), supporting and showing empathy (e.g., emotional support, being available), valuing and sanctioning work from home to facilitate well-being (e.g., prioritizing health, leading by example), and balancing individual and collective needs (i.e., flexibly adjusting leadership to employees’ individual needs and conditions while fostering a sense of community) [47]. Other studies on (ad hoc) virtual leadership during the COVID-19 pandemic referred to task-related behaviors (such as increases in meeting volume [31], setting a normal work schedule, creating a dedicated workspace, time management [53], setting team-specific rules [49]) and relationship-related behavior (such as setting aside time for building and sustaining relationships [58], being intentional about interactions, checking in with colleagues, engaging in informal team activities [53] and planning them in advance or initiating them strategically [59]).

Overall, according to current research, initial quantitative studies demonstrated the effectiveness of StaffCare also for digital, remote work contexts during the COVID-19 pandemic [9, 19–21]. Three of these studies analyzed StaffCare in comparison between office and home office [9, 20, 21]. Their findings indicated i.e. positive effects of StaffCare on employees’ well-being [20] as well as negative effects on employees’ mental exhaustion [21] and psychosomatic complaints [20] in both work contexts whereas two studies found lower effectiveness of StaffCare on employees’ engagement and job satisfaction in home office in comparison to office [9, 21]. Furthermore, two other studies conducted during the COVID-19 pandemic showed that, one, telework-oriented leadership (including StaffCare) affected teleworkers’ happiness well-being via strain by ensuring communication and information exchanges between teleworkers [60] and, two, that the positive relationship between StaffCare and follower health was stronger in times of crisis [61]. Lastly, according to current state of research, there are no comparable studies that have conducted a group comparison between leaders with and without prior experience with digital, remote collaboration in relation to healthy leadership.

### Preconditions of HoL in virtual teams

In a literature review on work design and remote working, Wang and colleagues [26] argued that prior to the COVID-19 pandemic, studies often considered work characteristics as moderators or mediators in the relationship between remote work and work experiences. Their third proposed approach, which was described as more appropriate for understanding remote working experiences for the period during the COVID-19 pandemic, no longer considered remote work as an independent variable but as a context, and thus work characteristics as antecedents in that context. The meaning of certain work characteristics such as social support was described as being shaped in the context of virtual work [26]. In addition, a recent review on leadership in virtual work settings outlined that work characteristics may have specific and sometimes diverse impacts on leadership effects and that these opposing mechanisms need to be taken into account [30].

Referring to our research question, regarding the enabling preconditions for HoL in virtual teams, the current state of research on personal, organizational, social and technical preconditions in this respect are discussed as follows. Leaders in the present study identified leaders’ characteristics and self-care, role modeling and leadership experience as personal preconditions for using HoL in virtual collaboration. To date, based on current research, only one qualitative study conducted prior to the COVID-19 pandemic exists that identified leader characteristics (leaders’ own ambition, too high demands and expectations towards oneself) as aggravating factor for using HoL in virtual collaboration [22]. In comparison to our results, in another qualitative study with leaders during the COVID-19 pandemic, technology literacy was most frequently reported alongside other relevant remote work skills and behaviors: being independent, communication, strong work ethic, ability to manage distractions, time management, personality (extraversion), supporting coworkers [53].

Similar to how leaders in the present study considered organizational preconditions for applying HoL in virtual collaboration, a qualitative study with virtual leaders from Germany also referred to a supportive management, flexible working conditions, an open-minded corporate culture and structural offers (e.g., occupational health offers or training courses) as supporting factors for leaders of virtual teams [22]. Other studies also indicated the relevance of organizational support for digital, remote leadership during the COVID-19 pandemic [7, 8, 31, 53]. Thus, with regard to management support, an interview study with leaders in Denmark pointed to the positive impact of crisis communication across the organization, although more concrete guidance for remote leadership was desired [8]. In terms of health-promoting working conditions, previous qualitative interview studies during the COVID-19 pandemic reported that high workload and less structured processes were perceived detrimental to remote leadership [7], and that agreements with management on working hours were perceived supportive in facilitating setting boundaries between work and leisure [31]. Additionally, a recent review on virtual leadership proposed a model on leadership in virtual settings illustrating qualifying factors of electronically mediated interaction (such as spatial or temporal dispersion) in the relationship between virtual leadership and employee outcomes [30]. In line with present results, institutional services were considered supportive for virtual leadership in other studies too: reimbursement for use of private electronic devices in home office as well as training on the use of new ways of working and digital communication including help by technical teams and management [31] (although in one study tools, advice and support was experienced more helpful at the beginning of the pandemic, but as it progressed, the support was lacking behind leaders’ experiences and not fitting their needs [8]). Further, similarly to our findings, an interview study with leaders from a healthcare organization in Canada found that corporate culture was a relevant enabler of remote work, which included supporting staff, positive perceptions of remote work, a value-focused organization, and embracing geographically dispersed teams [53]. However, independent of remote work context, studies on HoL investigated leaders’ job resources [32] and demands [48], organizational health climate [35, 36]), high-performance work practices or health-oriented human resources management strategies [37] as antecedents of HoL.

Consistent with our findings on social preconditions, other qualitative studies regarding virtual leadership before [22] or during the COVID-19 pandemic [7, 8] also identified social support by team, colleagues or own supervisor. On the one hand, self-organized teamwork, mutual support by the entire team [22], and a positive team spirit [8] were experienced as supportive (although one study pointed to a decrease in motivational team climate due to the implementation of virtual teamwork [7]). On the other hand, team members were also experienced as supportive by also paying attention to interpersonal conflicts or communication problems [22], showing commitment, dedication, and enthusiasm for the collaboration, and thus generating well-being through socialization among leaders [8]. Furthermore, social support by colleagues was described in terms of exchanges on good leadership practices and lessons learned (or by observing good leadership practices among colleagues [22]), inspirational and emotional support, and having a broad network in the organization [8]. In contrast, unhealthy leadership behaviors of leaders’ own supervisor were reported as another aggravating factor for applying HoL in virtual teams [22]. Previous studies on HoL beyond remote work research also assessed social support by colleagues [33], employees’ disclosure [62] and team health climate [34] as antecedents of HoL.

Although few leaders reported technical preconditions as a basis for HoL in virtual teamwork in the present study, other qualitative studies also pointed to technical problems in remote and digital collaboration such as poor internet connection, problems accessing data, inappropriate or inadequate home space and equipment [22, 31, 53]. Thus, it was reported that successful organizations in implementing working from home provided employees with remote data access solutions, upgraded hardware and software, made equipment available for home office use, provided IT support services, and offered ongoing support for leaders, IT professionals and technical teams to keep work running smoothly [31]. In addition, a quantitative study pointed to the impeding impact of ICT hassles on the positive relationship between StaffCare and employees’ mental health [19]. Another study suggested higher irritation levels among leaders experiencing more ICT hassles which was further linked to lower levels of StaffCare [63].Finally, while some of the studies referenced included leaders with varying levels of pre-pandemic experience with digital, remote collaboration [8, 31, 53], their findings were not presented or analyzed by prior experience. Accordingly, there are currently no comparable studies on preconditions of HoL that have conducted a group comparison between leaders with and without prior experience with digital, remote collaboration.

### Preventive measures for promoting HoL in virtual teams

Referring to our research question regarding preventive measures for promoting HoL in virtual teams, our results showed that all leaders named a variety of existing or needed behavioral preventive measures (e.g., further training for leaders and all employees) and structural preventive measures (e.g., support by management, improvement of technical equipment or general digitization processes). Similarly, a qualitative study with leaders from a healthcare organization in Canada that had little experience with managing remote work prior to the COVID-19 pandemic found that both behavioral and structural preventive measures were identified to support remote work in the future. On the one hand, the development of individual skills (e.g., managing virtually, maintaining rapport virtually, technology literacy, virtual soft skills, self-management) was suggested, and on the other hand, organizational changes (e.g., organizational culture shifts such as establishing trust in remote workers or “camera-on” culture, clear policies and processes, greater participation of employees, improved IT support and provision of appropriate technology and equipment) were proposed [53]. According to current research, there are hardly any further studies on prevention measures in virtual teamwork and leadership as well as on group comparisons between leaders with and without prior experience with digital, remote collaboration.

### Implications for future research

This qualitative study allows for hypothesis generation and yields many implications for future research. Overall, according to current state of research, to date there is no sound evidence on HoL implementation in digital remote collaboration [11, 18]. Therefore, future research should investigate, through experimental or qualitative studies, the StaffCare awareness of virtual leaders and how leaders can detect early warning signals via digital media. Moreover, future qualitative studies should also shed light on the employee perspective and examine how StaffCare is perceived by virtual team members, identifying challenges and differences to face-to-face StaffCare. Furthermore, quantitative longitudinal studies should survey the relationship between HoL and health- and work-related outcomes of virtual leaders and their team members in the future. In this regard, both external and self-reports should be considered, either by surveying dyads (leader and employee), entire teams (leader and team members) or in terms of a 360-degree feedback (e.g., leader, team members, supervisor, colleagues, customers).

Since there is still a great need for research on relevant influencing factors for virtual leadership and employee outcomes in general [11, 30], but also specifically on preconditions of HoL in traditional face-to-face as well as in digital remote work settings [18], it is recommended for future research to investigate diverse personal, organizational, social and technical preconditions in this context. This should include a differentiated and holistic examination of the specific conditions that leaders and employees need in order to effectively implement SelfCare and StaffCare in virtual teams. Further, it should be taken into account that characteristics of virtual teamwork may have partly diverse impacts on leadership effects [30] and that there cannot be a one-size-fits-all approach. Interventional studies are particularly recommended to study leadership effects post improvement of working conditions (e.g., via training programs on HoL or organizational culture change processes, see chapter on practical implications). In addition, future research should reveal how overarching contextual conditions such as the COVID-19 pandemic might differ from the post-pandemic period in their impact on virtual leadership.

### Implications for practice

Our findings highlight that in times of crisis such as the COVID-19 pandemic, in which abrupt workplace-related changes are imperative, challenges emerge for leaders in terms of their SelfCare and StaffCare. Moreover, it can be inferred from present exploratory results that prior experience with regular virtual or hybrid teamwork as well as adequate and comprehensive training prior for this activity may facilitate the implementation of HoL in times of crisis. Initial intervention studies on the effectiveness of HoL in traditional, face-to-face work settings indicate positive results of leadership interventions [64, 65] – although no other intervention studies exist yet for the virtual work context [18]. Furthermore, our results suggest that, in addition to enabling leaders and employees for HoL in virtual teams on a personal level, the organization also needs to adapt working conditions at the organizational, social, and technical levels. Thus, for successfully establishing a health-promoting leadership culture in an organization, a holistic perspective is required. For this purpose, a meta-analysis on workplace resources at the individual, the group, the leader, and the organizational levels in relation to employee well-being and organizational performance found that interventions at all of those four levels were effective. Accordingly, it was recommended that while organizations may engage at any level to effectively strengthen resources for employees, multilevel interventions should be preferred due to synergistic effects [66].

Overall, our findings on experiences, preconditions and preventive measures of HoL in virtual teams provide helpful insights for and recommendations for occupational health promotion in virtual teams. Thus, the first step is to perform a well-founded assessment of the organization through strong employee participation in order to identify the status quo and areas for action according to existing needs. After implementing interventions, a second step should take continuous, evidence-based evaluations into account. In this regard, organizations should note that, especially in times of crisis, ongoing, adapted organizational support is required according to actual and changing needs of employees. A list of possible areas for action are presented in Table 5.

**Table 5:** Practical implications for promoting HoL in virtual teams

Level of measure implementation	Behavioral preventive measures	Structural preventive measures
Personal	For leaders: • Empowerment of own health literacy: awareness for role model function, self-responsibility and health-oriented self-leadership • Further training and strengthening of leadership skills: e.g. on health-oriented, virtual/hybrid leadership (if applicable, specific further training on detecting early warning signs of mental illness and dealing with highly stressed employees) • Coaching For all employees: • Further training: needs-based qualification and skills reinforcement • Empowerment of own health literacy: self-responsibility and health-oriented self-leadership • Professional support in psychosocial employee counseling	For leaders: • Leadership programs for existing and new leaders • Instruments and leadership guidelines for orientation, e.g. annual employee interviews, 360-degree feedback tool For all employees: • HR development programs: transparent horizontal and vertical career paths
Organizational	• Use of measures for organizational culture development, e.g. training in dealing with failures (for building a culture of learning from failures) • Use of a wide range of institutional services, e.g. workplace health promotion offers	• Strategic alignment for cooperative collaboration between management, work council, human resources and change departments, and occupational health and safety • Support from management: role model function, long-term, preventive focus on health and well-being of the workforce, communication of appreciation and transparency • Establishing flexible, health-promoting working conditions • For organizational culture development: definition of corporate values, integration into corporate strategy • Participation of employees: regular employee surveys
Social	• Joint qualification and awareness measures in the team, team development measures • Formal and informal exchange formats for virtual collaboration among employees and leaders, e.g. best practice exchange programs • For communication problems and for dealing with conflicts: internal mediators or external experts for team consultation	• Promoting informal exchange: releasing budget for face-to-face meetings, enabling virtual teams to come together at regular times for social events
Technical	• Ongoing training for IT support services teams • Training for all employees when introducing new technical tools and workplace changes	• Provision of adequate IT support services • Improvement of general digitization processes, e.g. stable internet connection, lean communication channels, remote data access • Upgraded uniform technological equipment for all employees for offices and home offices • New technical solutions for digital, remote team and department development

### Strengths and limitations

Our study demonstrates several strengths. First, our study provides a valuable contribution to improving our understanding of the implementation of HoL in virtual collaboration – particularly in light of the massive worldwide increase of remote working during the COVID-19 pandemic. Moreover, using a qualitative research approach allowed for initial exploratory research findings in a new field of research [38]. Applied methods coincided and were appropriately selected for the subject. The PCI interview method enabled participants to speak openly about their experiences, combined with a structured interview procedure based on an interview guide. Conducting telephone interviews provided advantages in terms of research economy as well as flexibility in time and place to meet participants’ personal conditions. Consequently, anonymity during telephone interviews was perceived to be beneficial in building trust. Last, another strength lies in our sample distribution and size. The roughly equal distribution of the sample into two groups as to pre-pandemic experience with virtual or hybrid teamwork allowed for an exploratory qualitative group comparison. Our 16 conducted interviews were sufficient to achieve theoretical saturation [67].

Yet this study has some limitations. First, due to chosen recruitment strategy (snowball system), participant drop out could not be fully traced. According to qualitative research, no generalizability of research results but hypothesis generation was intended. Nevertheless, it must be mentioned that any external influences during our telephone interviews, e.g. due to technical malfunctions in individual interviews, could not be controlled – although no consequences for the course of the interview were determined. Furthermore, our results only represent a leader perspective. In addition, self-selection bias and socially desirable response behavior could not be entirely avoided. It remains questionable whether the participants also assign the same importance to health apart from the interview situation or whether their self-reports match their actual leadership behavior. When interpreting the explorative group comparison, it is important to note that quantitative data of radar charts are not comparable without limitations. Despite using an interview guide, participants were free to decide what they wanted to report on the questions. Thus, not all participants commented on all categories. In addition, although qualitative data collection and analysis methods were carefully applied as well as critically reflected by the interviewer by means of postscripts, multiple realities (i.e. influences through researcher’s perspective) cannot be completely eliminated in qualitative research [39, 42]. Last, it is necessary to consider the context of study conduct when interpreting results. Although it was intended to capture experiences with (ad hoc) leadership during the COVID-19 pandemic, dynamic pandemic trajectories over time must be considered. The data were collected in 2021, a period when a great deal of transition and uncertainty still existed in many organizations regarding virtual or hybrid collaboration and leadership [26, 68]. It can be discussed whether a habituation effect occurred in later stages of the pandemic, so that pre-pandemic experience with virtual or hybrid teamwork no longer made a decisive difference for leaders.

## Conclusions

Given the increased digitization and workplace-related changes during the COVID-19 pandemic, the aim of this study was to examine the experiences of virtual leaders during the COVID-19 pandemic and to identify preconditions and preventive measures for promoting HoL. By using a qualitative research approach, we illustrated that leaders, regardless of pre-pandemic experiences with virtual leadership, faced diverse challenges in implementing HoL in (ad hoc) virtual teamwork during the COVID-19 pandemic. Overall, we found that implementing SelfCare and StaffCare in virtual teamwork is very challenging and complex – apart from leaders’ own awareness and motivation, they have to consider many external influences to be able to apply appropriate leadership behavior. Thereby, our study presented initial empirical findings for a holistic approach to HoL implementation in virtual teams, considering beneficial preconditions on personal, organizational, social and technical levels. This study provides a basis for future research, including longitudinal and interventional studies to analyze the causal relationships between preconditions, HoL in virtual teams and health-related outcomes. In particular, we recommend a holistic research perspective in order to understand the complex, contextual interdependencies of leadership. In practice, a holistic perspective is also recommended, which requires a differentiated implementation and evaluation of multilevel behavioral and structural preventive measures adapted to the needs of employees and the organization in order to achieve effective change in organizations.

### Electronic supplementary material

Below is the link to the electronic supplementary material.

**Supplementary Material 1:** COREQ checklist

**Supplementary Material 2:** Interview guide

**Supplementary Material 3:** Coding tree

**Supplementary Material 4:** Interview quotes

## Data Availability

The data analyzed during the current study are not publicly available due to German national data protection regulation. The data used and analyzed during the current study are available from the corresponding author on reasonable request.

## Abbreviations

SelfCare: Health-oriented self-leadership
StaffCare: Health-oriented employee leadership
HoL: Health-oriented leadership
