# Supplementary material for: “That was one of my most difficult and biggest challenges”: experiences, preconditions and preventive measures of health-oriented leadership in virtual teams – A qualitative study with virtual leaders
Source: BMC Public Health. 2024 May 17;24:1338. doi: 10.1186/s12889-024-18800-7 (PMC11102273; doi:10.1186/s12889-024-18800-7)
Supplement: Supplementary file 2 — Supplementary Material 2: Interview guide [file 12889_2024_18800_MOESM2_ESM.pdf]

Supplementary material for “That was one of my most difficult and biggest challenges”: Experiences, preconditions and preventive measures of health-oriented leadership in virtual teams – A qualitative study with virtual leaders. (Efimov et al.)

Supplementary material 2: Interview guide

Job-related and socio-demographic information will be taken prior to main interview questions.

1. What are the challenges for (ad hoc) health-oriented leadership in virtual teamwork in times of the COVID-19 pandemic?
   - 1.1. As a leader, were you able to keep an eye on health at work during the pandemic? Both of yourself and of your employees?
   - 1.2. Despite the COVID-19 pandemic, how have you managed to in-corporate health-oriented leadership into your work?
2. What influences you in your health-oriented leadership behavior or in your motivation to lead in a health-oriented way?
3. What measures are needed to promote the use of health-oriented leadership in virtual teamwork?
   - 3.1. Which measures were found to be helpful?
