# Supplementary material for: “That was one of my most difficult and biggest challenges”: experiences, preconditions and preventive measures of health-oriented leadership in virtual teams – A qualitative study with virtual leaders
Source: BMC Public Health. 2024 May 17;24:1338. doi: 10.1186/s12889-024-18800-7 (PMC11102273; doi:10.1186/s12889-024-18800-7)
Supplement: Supplementary file 3 — Supplementary Material 3: Coding tree [file 12889_2024_18800_MOESM3_ESM.pdf]

Supplementary material for "That was one of my most difficult and biggest challenges": Experiences, preconditions and preventive measures of health-oriented leadership in virtual teams – A qualitative study with virtual leaders. (Efimov et al.)

Supplementary material 3: Coding Tree

Codes	Categories	Aggregated categories
Feasibility of implementing SelfCare in home office during COVID-19 pandemic	Feasibility of implementing SelfCare in home office during COVID-19 pandemic	Implementation of SelfCare in home office during COVID-19 pandemic
Challenges for SelfCare implementation	Challenges for SelfCare implementation	
Feasibility of implementing StaffCare in virtual teams during COVID-19 pandemic	Feasibility of implementing StaffCare in virtual teams during COVID-19 pandemic	Implementation of StaffCare in virtual teams during COVID-19 pandemic
Fewer cues in virtual communication	Challenges for StaffCare implementation	
Limited possibilities and quality of informal exchange		
Challenges maintaining or building proximity to employees		
Private challenges of employees		
Consideration of employees' needs and competence	StaffCare behaviors to cope with challenges	
Proactive communication with employees		
Redesigning team meetings		
Redesigning one-on-one meetings		
Characteristics of the leader	Personal preconditions	Preconditions of HoL in virtual teams
Self-care of the leader		
Leader as role model		
Leadership experience		
Supportive management	Organizational preconditions	
Supportive, open-minded corporate culture		
Health-promoting working conditions		
Wide range of institutional services		
Employee-oriented, supportive work council		

Social support by team<br>Social support by colleagues or own supervisor	Social preconditions	
Stable internet connectivity<br>Sufficient technical equipment<br>IT support by organization	Technical preconditions	
Further training for leaders<br>Further training for all employees	Behavioral preventive measures	Preventive measures for promoting HoL in virtual teams
Support from management<br>Improvement of technical equipment and general digitization processes	Structural preventive measures	
