# Supplementary material for: “That was one of my most difficult and biggest challenges”: experiences, preconditions and preventive measures of health-oriented leadership in virtual teams – A qualitative study with virtual leaders
Source: BMC Public Health. 2024 May 17;24:1338. doi: 10.1186/s12889-024-18800-7 (PMC11102273; doi:10.1186/s12889-024-18800-7)
Supplement: Supplementary file 4 — Supplementary Material 4: Interview quotes [file 12889_2024_18800_MOESM4_ESM.pdf]

Supplementary material 4: Additional interview quotes

Chapter: Implementation of SelfCare in home office during COVID-19 pandemic

> *"But in this entirely digital environment [...] I simply allowed myself far too few breaks to breathe in this highly structured professional schedule. After three or four months, I simply forced myself to take a lunch break, to go outside, no matter what weather it was, to get some fresh air, to clear my head, so that I didn't rush from one team meeting to the next and from one call to the next - like a hamster - but to schedule more time for myself to relax. [...] Because you need to be present in a completely different way. When you have a lot of topics to deal with and are involved in developing them, it's different from just sitting casually in a conference room at the office and letting things happen to you and just listening. That is much less. So, the concentration and also the preparation of the topics is more intensive." [Participant #14, age ≥ 61 years, team lead]*

Chapter: Feasibility of implementing StaffCare

> *"Yes, I think it really has a lot to do with how you interact with your employees. […] And it's not that communication has disappeared, it's just different." [Participant #13, age 31-40 years, team lead]*

> *"I believe that if someone can't lead face-to-face in a healthy way, they probably can't in digital either." [Participant #16, age 31-40 years, team lead]*

Chapter: Challenges for StaffCare implementation

Fewer cues in virtual communication

> *"And I believe that a great deal is now possible. As I said, using camera on, you can notice quite a lot. Nevertheless, I would still say that it makes a difference if you meet in the office. There's something that I still believe so far (laughs), that it can't just be compensated digitally so easily. That's the energy you feel when you're in the room. Humans are more than just mind-controlled, and you perceive a lot through your senses, which is what people always mean by perception or awareness." [Participant #6, age 51-60 years, head of department]*

> *"Yes, so I would say it made me, I simply underestimated that over the first few weeks too, how difficult it is to assess how an employee is doing. So, I have an example. A*

colleague changed from another team to my team during that time and had to familiarize herself with the work. She made an extremely good impression to me, was somehow very positive the whole time, and also put up a good front. And at some point, I gave her interim feedback after two months and said: 'You're doing a great job' and then it came out that she worked until eight o'clock every night and, let's say, actually couldn't cope at all and was far too overworked and that she wasn't doing well with this situation either. And I didn't notice that at all. So […] through the meetings that you have, I simply didn't notice that and afterwards I was, fortunately that was very early on, I was very sensitized, actually towards all employees, that you simply have to listen a lot, a lot more, because in case of doubt you just don't see it in the person anymore when you only see each other through a camera. […] But I think if I were in the office, I would have noticed it more quickly. Because, if you ask employees, they often, depending on their character, put a good face on the matter and say: 'I can do it and I'm fine. So, before you open up, even to your supervisor, you don't do this at the slightest sign of trouble, I'd say." [Participant #4, age 31-40 years, team lead]

"So, it's not so easy to pay attention to people's physical health, obviously, because I can't see how they're sitting in front of the screen. Does he have the screen adjusted correctly? Does he actually have a warm water bottle on his stomach because of stomach pains or something like that? I can't assess such things. The only thing that really helps is open conversation, and fortunately my employees are honest with me so that I can rely on them telling me if they're not feeling well." [Participant #4, age 31-40 years, team lead]

Limited possibilities and quality of informal exchange

"Yes, and you can't create any incentives. In the past, colleagues came to the office in the evening because we have a really cool office here with a cool view, and they met here for a game night [...]. And of course, as an employer, you can encourage that. Of course, you can say: 'There are drinks in the fridge and you can use them if you want'. So of course, you can make offers that increase the chance that there are social bonds beyond work itself, which is also good for added value. But if they're not there, what are you going to do? Of course, you can send everyone a beer to their homes, which is what we did once and then met virtually for a beer on Friday afternoons. That's so lame after the second time. Yes, the possibilities are also extremely limited and that's just, yes you just have to live with the fact that you have to manage without it. I'm rather

> *frustrated at this point, but I can't think of any solutions.” [Participant #2, age 51-60 years, management]*

Challenges maintaining or building proximity to employees

> *“Well, it works, but you have to find the right approach. And then via Zoom [a video conferencing system], where it's difficult to build up a personal relationship anyway, I think you need a bit more time and calm in between.” [Participant #16, age 31-40 years, team lead]*

Private challenges of employees

> *“Of course, everyone notices the change in their daily environment. [...] That's why we very consciously said right at the beginning that we won't close the office, but that we are grown-up, self-responsible and self-organized, and whatever else we can think of. Anyone who thinks they can work better in the office than at home is welcome to come to the office. We make sure that this is in line with regulations and that people don't get infected here, and we have enough space, and there are some who say: 'I have two small children at home and I can't work there'. Or: 'If I'm at home for five days, then I just run around in sweatpants and don't shave anymore.' So, who realize by themselves that this is not good for them, that they need this regular getting out of the house. Some colleagues who live in a three-room apartment and say: 'I don't want to sit at the kitchen table for another eight hours in this three-room apartment and work, but I want to get out.' And that also has something to do with health promotion, so of course we give them the opportunity to work here, there's no question about that.” [Participant #2, age 51-60 years, management]*

Chapter: StaffCare behaviors to cope with challenges

Consideration of employees’ needs and competence

> *“Yes, it has been particularly difficult for people who had a leadership position, following a very, very old model. For them, controlling work means going through the offices and checking whether people are working. For them, times have become very, very difficult. [...] Yes, and transparency is an issue that is very much needed when you only work remotely.” [Participant #11, age 51-60 years, head of department]*

> *“And that's what I mean by personal circumstances and the setup each individual has. And as a leader, you can of course respond to this to some extent and also be*

supportive, but above all, I think you have to guide employees a bit in helping themselves." [Participant #13, age 31-40 years, team lead]

Proactive communication with employees

"Yes, you have to be very careful, because colleagues who already tend to withdraw and [...] don't want to be the center of attention, have withdrawn even more during this pandemic phase. That means [...] I had to invest more in order to keep up with them so that they don't get completely lost in this situation." [Participant #14, age ≥ 61 years, team lead]

Redesigning team meetings

"Maybe we'll do another two-day [team meeting] in June, it depends a bit on the topics, but it's very exhausting to spend two whole days virtually, via video [...], just meeting this way. That's why we've shortened it to one day, and we're also applying appropriate meeting etiquette with appropriate breaks. Otherwise it becomes very exhausting." [Participant #10, age 51-60 years, team lead]

"Well, of course I'm also in meetings every day. But I'm not one of those people who have their calendar timed from morning to night. [...] So I also consciously schedule days where I have almost no meetings at all, or none at all." [Participant #2, age 51-60 years, management]

"Apart from that, what we've done is actually, that's what I've noticed, actually introduced a lot of meeting rules. Digital meeting rules. Because at the very beginning it was just, it's still a bit difficult sometimes, but at the very beginning, depending on which group you were in, it was really hard. So, if you're sitting in a group with only technicians, for example, then ideally, they have turned off the camera and are 'muted' for the entire meeting. So that was very tough at the beginning. So, there were very, very many who said very little, were permanently 'muted' and had their cameras off for a long time. And today that has almost disappeared. Today, there is almost no one who has the camera off." [Participant #15, age 31-40 years, team lead]

"At the beginning of the pandemic, we hardly ever met in person. Between the first and second lockdown, there was a period of gradual reductions, and we held hybrid meetings. In one meeting room, four or five team members were on site, and the rest were connected remotely via teams. Despite technical obstacles, this actually worked quite well." [Participant #8, age 31-40 years, team lead]

> *"We have introduced new team meetings. So up until now, it was just once a week. And we have added two more. Another one focused on content, where we organized content a bit differently, and one where it's just about socializing, where you can just chat with each other." [Participant #6, age 51-60 years, head of department]*

> *"Exactly, so what we've done is that at longer intervals, but we've done it, we've also simply held virtual team evenings. But I asked my team beforehand whether they wanted to do that. Simply because I was a bit worried that the team spirit might get lost if everyone lives or works alone. That's what we did. It was actually really nice. Everyone said beforehand: 'Well, let's see how it turns out virtually. You already sit in front of your computer all day long.' But then it went extremely well. We do this every six weeks or so, and we call each other in the evenings and just talk privately for a bit. Because that's actually something that's neglected a little bit, because otherwise you just talk to each other in the office and get to know a lot about people, and otherwise you only get to know that in bilateral conversations. And we introduced a regular team meeting. We didn't have one before, because we were all sitting in one office and somehow the exchange was already huge. And then we always had a regular team meeting once a week, but we have an extremely large number of meetings where we also see each other." [Participant #4, age 31-40 years, team lead]*

Redesigning one-on-one meetings

> *"Well, I have an additional weekly meeting with each one, a digital meeting. And one thing has also become very clear over time: Camera on. Well, that was or still is the case with some people, so in some cases the camera doesn't work or they don't have one at all or some people don't want it anyway, and you can't force anyone to use it. But that has really become clear in our team, that it makes a big difference whether you have the camera on or not." [Participant #6, age 51-60 years, head of department]*

Chapter: Personal preconditions

> *"A sound knowledge of people, being able to assess people, approaching people. Also, to have trust. If you don't have that trust, then at some point you end up with micromanagement and control issues, and that puts a strain on you and other colleagues as well. So, trust plays a very important role. And a certain knowledge of human nature." [Participant #11, age 51-60 years, head of department]*

> *"First and foremost, I think we need leaders who are open to trying out new things and are then perhaps also open to giving employees a bit more space. Because I believe that with digital, those who were already quiet are perhaps even quieter. Especially in a team meeting at Zoom [a video conferencing system], you can't just interrupt someone. You have to be very attentive with your team. I think the first thing you need is a willingness on the part of the leader to tackle these challenges." [Participant #16, age 31-40 years, team lead]*

> *"You also have to be able to take care of yourself and stay physically fit, and sometimes even have enough patience to meet the demands and just be there. (...) If you are very insecure and cannot convey security and confidence, then I think it becomes difficult." [Participant #14, age ≥ 61 years, team lead]*

> *"Basically, there are three buzzwords that are always widely used: Communication, transparency and trust. So, there are three things you have to practice. You need to do more than just say, 'Here, trust each other.' You have to set an example." [Participant #8, age 31-40 years, team lead]*

Chapter: Organizational preconditions

> *"For example, we agreed jointly that certain tools should be used for task management and for collaboration, but then only two of us use them. And I have to say that this is also a 'crap rolls downhill' issue, because the managing director himself doesn't do it and set a good example. This is another classic leadership issue." [Participant #13, age 31-40 years, team lead]*

> *"Fortunately, we also have a management that actually practices this, that doesn't just demand it from the top down, but actually practices it too. And that is another big step in moving towards change." [Participant #8, age 31-40 years, team lead]*

> *"For example, we work internationally a lot and that's just great with people from three different continents, we had that the other day, being together in a workshop. That's just great, that's fun. That's the global world, the small global village in its purest form, so to speak, so that's really good." [Participant #6, age 51-60 years, head of department]*

> *"These are quite trivial, these ergonomic issues and of course occupational safety, I need a proper table, I need a proper chair, I need quiet when working. And these are preconditions that we check very carefully before the pandemic, i.e. the working conditions at home by means of a questionnaire. And we checked: Do you have all the prerequisites that are at least so favorable that you can work like this? And I have to make the same requirements for myself. I simply have to know that I have a proper workplace in order to be able to work in this way. Regardless of whether I am a leader or not." [Participant #10, age 51-60 years, team lead]*

> *"Of course, there are a lot of requirements at the moment. And in some areas, we have a lot to do, yes. That is definitely an issue. As I said, and the other thing is speed. [...] I think this is the case with some leaders, who make it easy for themselves and just pass it on, like throughput heaters. And they just pass that on to the employees and say, "Here, you have to implement it." And they don't filter that at all and they don't manage that." [Participant #1, age 51-60 years, head of department]*

> *"What also helps us is some kind of simple training offer from our HR development team, which is currently putting everything on the marketplace that has to do with stress management and resilience and virtual leadership, coaching, etc. All employees can book themselves into various training courses and seminars. [...] And of course, I'm very happy to offer these things, even if it costs money, because I know that it's a good investment." [Participant #5, age 31-40 years, team lead]*

> *"Whereas in the company where I am now, there is a very strong work council, which for a long time strongly prevented the introduction of such media and also did not or could not create the possibilities for employees to work together more easily via digital media." [Participant #7, age 41-50 years, management]*

Chapter: Social preconditions

> *"And then my team was also ready to try things out and follow new approaches. That certainly helped a lot, too." [Participant #9, age 51-60 years, head of department]*

> *"But the team rewards me with a lot of commitment and joie de vivre and very great communication and also transparency towards me." [Participant #8, age 31-40 years, team lead]*

"It is possible, but the employees' individual responsibility is of course even more challenged here than in the office." [Participant #8, age 31-40 years, team lead]

Chapter: Technical preconditions

"If you look at our operational activities for our customers, we were very well prepared because we had already started beforehand. In other words, we were able to increase our capabilities and soon had 80-90% of our employees in home offices. We were rather poorly equipped in terms of the question: How can we maintain social contacts? What about the webcam? How about access with Microsoft Teams, which I mentioned earlier, in order to have chat functions, etc. pp. We were rather poorly equipped in that respect, and in some cases, we are still poorly equipped. That also has to do with overloading our network. Of course, you can imagine that if all 30,000 employees turn on the video camera and then have a casual meeting, that's also a problem. So far, we haven't been able to ensure that everyone has a video camera. [...] In our company, leaders have the equipment, while non-leaders usually do not. And you simply notice big differences. Whether you hold a blind telephone conference, in quotation marks, or whether it's a video conference. That goes along with, how is the attention? And that's also something that you notice, of course, when you speak off-camera, in quotation marks (laughs), you have the feeling, but I think (laughs) actually proven to have less attention and it's being done more alongside. That's why you're usually done much faster, because of course there's no whispering left and right." [Participant #9, age 51-60 years, head of department]

"On a personal level, I had planned to do that for quite some time anyway, but here in our house, we live on the countryside, I did everything I could to stabilize the internet connection. I didn't have a cable in the basement, and now I've had it installed here and so, I've also invested a little bit. Because that is one of the most important prerequisites, I would say, for having stable internet. A stable connection is simply the key." [Participant #6, age 51-60 years, head of department]

Chapter: Behavioral preventive measures

"I think it's important, as I've seen it in the past, for all leaders to be made aware of these issues and given the tools to deal with them. How do I deal with difficult situations? How do I approach employees? How do I recognize that there might be a

problem? It doesn't matter whether it's in the team or with the employee himself. Where can I get help? What should I do better on my own, and where should I do it with one of our trained HR people, for example, who are aware of this in a different way? How do I approach such issues?" [Participant #14, age ≥ 61 years, team lead]

Chapter: Structural preventive measures

"I can only recommend to others: Make sure that every single person in the company knows what share they have in creating value. I believe that this is the ultimate key to success. [...] This is an extremely intrinsically motivating element, which I believe leads to a high level of satisfaction for each individual. And if I do a lot of controlling, i.e. classic management, and tell people what they have to do and what they don't have to do [...], then I deny them precisely their competence to be able to decide things for themselves [...]. This will not go down well in the long run. [...] I think this is extremely important, also for health." [Participant #2, age 51-60 years, management]

"The first thing that comes to mind is the topic of 'better technical equipment', i.e. webcam. The next thing that goes hand in hand with that is, of course, the telephone system, telephone quotas, etc." [Participant #9, age 51-60 years, head of department]

"I don't have a solution for that now, but what I have missed during that time, and we often discussed this in the team, was that we always thought we still have a really good team bond, you feel like you know what's going on with the others. Somehow, we found a good solution for us. But what we don't have at all anymore is a department feeling. Because, of course, you have to deal with the team all the time, we have regular meetings together, I talk to almost every one of my employees every day. But in the department, this has been completely lost. And I don't really have a solution for that, but that's a great pity, because we do have a digital meeting, but 30 people are too many for that. I also suggested that we use breakout sessions again, which we have done now, that we get together again in small groups, so to speak, but with others. I think coffee calls were a good opportunity. I think that's something that could be solved even better if such a situation were to continue for a longer period of time, i.e., working digitally from a distance." [Participant #4, age 31-40 years, team lead]
